# Evaluation of the Leaves and Seeds of Cucurbitaceae Plants as a New Source of Bioactive Compounds for Colorectal Cancer Prevention and Treatment

**DOI:** 10.3390/nu16234233

**Published:** 2024-12-07

**Authors:** Mercedes Peña, Ana Guzmán, Cristina Mesas, Jesús M. Porres, Rosario Martínez, Francisco Bermúdez, Consolación Melguizo, Laura Cabeza, Jose Prados

**Affiliations:** 1 Institute of Biopathology and Regenerative Medicine (IBIMER), Center of Biomedical Research (CIBM), University of Granada, 18100 Granada, Spain; mpenacontreras@ugr.es (M.P.); cristinam@ugr.es (C.M.); jcprados@ugr.es (J.P.); 2 Instituto de Investigación Biosanitaria de Granada, ibs.GRANADA, 18012 Granada, Spain; 3 Cellbitec S.L., Scientific Headquarters of the Almeria Technology Park, University of Almeria, 04128 Almeria, Spain; ana.guzman@cellbitec.com (A.G.); francisco.bermudez@cellbitec.com (F.B.); 4 Department of Physiology, Institute of Nutrition and Food Technology (INyTA), Biomedical Research Centre (CIBM), Sport and Health Research Institute (IMUDS), University of Granada, 18016 Granada, Spain; jmporres@ugr.es (J.M.P.); rosariomz@ugr.es (R.M.); 5 Department of Anatomy and Embryology, Faculty of Medicine, University of Granada, 18071 Granada, Spain

**Keywords:** cucurbitaceae, *Cucurbita*, extracts, colorectal cancer, antitumor, antiangiogenic, antioxidant, chemopreventive

## Abstract

Background/Objectives: The Cucurbitaceae family represents an important source of bioactive compounds with antioxidant, antimicrobial, anti-inflammatory and antitumor activities. This study aims to investigate the potential application of Cucurbitaceae leaves and seed extracts to prevent and treat colorectal cancer (CRC). Methods: Four extracts (ethanol extracts and protein extracts and hydrolysates) from the leaves and seeds of cucurbits were tested in T-84, HCT-15 and HT-29 CRC cells. The antitumor, antiangiogenic, antioxidant and chemopreventive potentials and bioactive composition of the active extracts were characterized. Results: Cold ethanolic extracts from the leaves and seeds of two interspecific *Cucurbita* genera (CLU01002 and COK01001) exhibited potent antiproliferative, specific and non-hepatotoxic activity against CRC cell lines, with a slight synergistic effect in combination with oxaliplatin. This antitumor activity was related to G_2_/M cell cycle arrest, the extrinsic apoptosis pathway, cytokinesis inhibition and autophagy. The extracts also inhibited tumor clonogenicity and angiogenesis, and modulated cancer stem cell (CSC) gene expression, as well as expressing antioxidant and chemopreventive cellular capabilities. Finally, phenolic and cucurbitane-type triterpenoid compounds (pengxianencins and cucurbitacins) were tentatively identified in the active extracts by UPLC-MS analysis and bioguided fractionation. Conclusions: Extracts from leaves the and seeds of two interspecific *Cucurbita* genera (CLU01002 and COK01001) may contribute to the improvement of prevention and treatment strategies for CRC patients.

## 1. Introduction

The Cucurbitaceae family, also called cucurbits, is composed of approximately 1000 species that are globally distributed and highly valued as a food source as well as for their applications in ethnoveterinary practice, cosmetic industry and both traditional and modern medicine [1]. The Cucurbitaceae family could be a promising source of functional foods and new therapeutic agents due to their demonstrated antioxidant, antidiabetic, antineoplastic, anti-inflammatory and antimicrobial properties which have been related to phenolic compounds (caffeic acid, chlorogenic acid, luteolin, apigenin, epigallocatechin-3-gallate, gallic acid, quercetin, quercitrin, isoquercetin, etc.), triterpenoids (cucurbitacins), alkaloids, polysaccharides, steroids, peptides and carotenoids (in fruits, roots, leaves and seeds) [1,2,3].

Antioxidant molecules related to cancer prevention have been identified in some cucurbits plants such as bitter melon (*Momordica charantia*), pumpkin (*Cucurbita maxima*), chayote (*Sechium edule*) and *Citrullus colocynthis* [4,5,6,7]. Conversely, many extracts from cucurbits species including *M. charantia*, *M. cochinchinensis* and *Trichosanthes kirilowii* were found to be cytotoxic in breast and ovarian cancers [8], lung cancer [9], prostate cancer [10] and leukemia [11]. This antitumor potential has been frequently associated with cucurbitacins, cucurbitane-type triterpenoids with a bitter taste and high toxicity in in vitro and in vivo cancer models, such as cucurbitacin B, D, E, I and IIa [12,13,14,15,16,17].

There is concrete evidence that, in colorectal cancer (CRC), the third most commonly diagnosed cancer in the world and second leading cause of cancer-related mortality [18], Cucurbitaceae plant extracts and bioactive compounds (i.e., cucurbitacins) exhibit a significant antitumor potential [19,20,21]. These results support that the development of functional foods or nutraceuticals and their combination with conventional anticancer treatments may induce a beneficial effect due to their antiproliferative, antimetastatic, antiangiogenic, antioxidant and anti-inflammatory properties [22,23]. This combined therapy could overcome some causes of cancer treatment failure such as drug resistance and the non-specificity of conventional treatments based on the use of chemotherapy, radiotherapy and molecular-targeted therapy [24]. In fact, a number of phytochemicals, including polyphenols, flavonoids, alkaloids and terpenoids, have demonstrated efficacy in preventing and treating CRC, even in clinical trials in combination with standard treatments (e.g., curcumin and silymarin) [25,26,27]. Moreover, some plant-derived bioactive compounds, including curcuminoids, resveratrol and quercetin, have recently been “Generally Recognized As Safe” by the US FDA for their use as dietary supplements, but they have not yet been regulated as drugs [28].

The aim of this study was to evaluate the bioactive potential of four different extracts from eight cucurbit plants, mostly from the *Cucurbita* genus, in CRC prevention and treatment and to tentatively determine their composition. Extracts from cucurbit varieties CLU01002 and COK01001 showed significant antitumor activity, alone or in co-administration with cytotoxics classically used against CRC (oxaliplatin). In addition, a relevant antiangiogenic, antioxidant and chemopreventive effect was demonstrated. These results support that the Cucurbitaceae family could be a source of bioactive compounds with pharmacological activities related to CRC prevention and treatment.

## 2. Materials and Methods

### 2.1. Cell Culture

The following human CRC cell lines, T-84, HT-29 and HCT-15, and the human hepatocellular carcinoma cell line HepG2 were acquired from the American Type Culture Collection (ATCC; Rockville, MD, USA). The human non-tumor colon cell line CCD-18Co was provided by the Centre for Scientific Instrumentation of the University of Granada (CIC-UGR, Granada, Spain). The cells were cultured in DMEM (Sigma-Aldrich, St. Louis, MO, USA) supplemented with 10% heat-inactivated fetal bovine serum (Gibco, Madrid, Spain) and 1% of penicillin-streptomycin antibiotic (Sigma-Aldrich) in a humidified atmosphere at 37 °C and 5% CO_2._

### 2.2. Plant Material

The leaves from plants of the *Cucurbita* genus (*C. pepo*, *C. moshata*, *C. maxima*, and interspecific hybrids) and from *Sechium edule* (chayote), as well as seeds from the two interspecifics from *Cucurbita* genus, CLU01002 (CLU) and COK01001 (COK), were generously provided by Agrointec Solutions S.L. (Almeria, Spain). For subsequent extraction methods, the leaves were lyophilized, and seeds were grounded to reduce the particle size.

### 2.3. Cold Ethanolic and Reflux Ethanolic Extracts

Cold ethanolic and reflux ethanolic extracts were collected following the protocols of Kapravelou et al. (2015) [29] and Martinez et al. (2024) [30] with small modifications. In brief, about 2.5 g of leaf and seed powder was subjected to cold or reflux ethanolic extractions. For cold ethanol extraction, the samples were extracted twice with a hydroalcoholic solution (50:50:0.25 ethanol: water: 12N HCl) at 4 °C, pH 2, and under agitation and a nitrogen atmosphere for 30 min. The supernatants were collected using centrifugation (3500 rpm, 10 min, 4 °C), and mixed. For reflux extraction, samples were treated with a hydroalcoholic solution (50:50:0.40 ethanol–water–12N HCl) using a two-stage (immersion + washing) Soxhlet extraction technique (SER 148 semiautomatic solver extractor, VELP, Usmate, Italy). Following the extraction procedure, ethanol was removed from the extracts by evaporation for 75 min using a vacuum evaporator (Savant DNA120 SpeedVac Concentrator, Thermo Fisher Scientific, Waltham, MA, USA) before storage at −20 °C.

### 2.4. Protein Extracts and Protein Hydrolysates

Protein extracts and protein hydrolysates were prepared in accordance with the methodology described by Kapravelou et al. (2013) [31]. In brief, powdered leaves were submitted to a sequential aqueous extraction twice (30 min, 33 °C and pH 8.8). After the extraction process, the samples were centrifuged, and supernatants were collected and mixed. Following centrifugation, the combined supernatants were heated (47 °C, 20 min) under stirring and 100 mM CaCl₂ and MgSO₄ solutions were added in a 1:100 *v*/*v* ratio. Subsequently, the aqueous protein extracts were subjected to hydrolysis treatment with proteases from *Bacillus licheniformis* (0.3 Anson Units/g leaf protein, 47 °C, pH 8.8, 30 min) and *Aspergillus oryzae* (100 LAPU/g leaf protein, 47 °C, pH 8.8, 30 min). The protein extracts and hydrolysates were lyophilized, resuspended in purified water and heated at 95 °C for 10 min before use in cell cultures.

### 2.5. Cell Viability Assay

The following cells per well (c/w) plate were seeded in supplemented DMEM: T-84 (5 × 10^3^ c/w), HT-29 (15 × 10^3^ c/w), HCT-15 (6 × 10^3^ c/w) and HepG2 (5 × 10^3^ c/w) in 48-well plates (300 μL), and CCD-18Co (2 × 10^3^ c/w) in 96-well plates (150 μL). The cells were incubated with the extracts at increasing doses for 72 h. A sulforhodamine B (SRB) cytotoxicity assay [32] was performed and OD was measured at 492 nm with a BioTek800TS microplate reader (Agilent, Santa Clara, CA, USA) to calculate the percentage of proliferation relative to untreated cells (RP, %).

### 2.6. Synergistic Effect Analysis

The T-84 cell cultures in the 48-well plates (5 × 10^3^ c/w) were exposed to varying concentrations of the extracts in combination with 5-fluoruracil (5-FU) and oxaliplatin (OXA) for 72 h, and cell proliferation was determined by the SRB assay [32]. Synergy scores were calculated using the HSA (highest single agent), Bliss, Loewe and ZIP (zero interaction potency) [33] models, implemented with SynergyFinder Plus (Helsinki, Finland) [34].

### 2.7. Cell Cycle and Apoptosis Flow Cytometry Analysis

For the cell cycle assay, T-84 cells were plated in 12-well plates (5 × 10^4^ c/w) and treated for 48 and 72 h after a 24 h incubation with serum-free DMEM to synchronize the cell cycle. Finally, the cells were detached, fixed with 70% ethanol and stained with a PI/RNAse solution (Immunostep, Salamanca, Spain), according to the provider’s specifications. For the apoptosis assay, T-84 cells were seeded in 6-well plates (1 × 10^5^ c/w), and exposed to the treatments for 48 h. Then, the cells were detached and stained with annexin V-FITC and PI from the FITC Annexin V Apoptosis Detection Kit (Immunostep), following the manufacturer’s instructions. An FACS Canto II flow cytometer (Becton Dickinson, Franklin Lakes, NJ, USA) was used for the flow cytometry analysis.

### 2.8. Lysotracker Labeling

T-84 cells were grown in 8-well chamber slides (1.5 × 10^4^ c/w) and treated with extracts for 24 h. Then, the cells were stained with 50 nM LysoTracker Red DND-99 (Thermo Fisher Scientific) for 30 min and with Hoechst 33342 (1:1000) for 5 min. They were observed by fluorescence microscopy (Leica DM IL LED, Leica Microsystems, Wetzlar, Germany), and fluorescence was quantified using the ImageJ software (version 1.53k).

### 2.9. Immunofluorescence Assay

Cells were seeded in 8-well chamber slides (1.5 × 10^4^ c/w) and incubated with the extracts for 24 h after cell attachment. Immunofluorescence staining was performed according to the modified protocol of Mesas et al. (2021) [35]. In brief, cells were fixed, permeabilized, blocked and incubated with a primary mouse anti-α-tubulin mAb (T9026, Sigma Aldrich, 1:250) and a secondary goat anti-mouse antibody, Alexa-Fluor^TM^ 488 (A11001, Invitrogen, Thermo Fisher Scientific, 1:2000). Nuclei were stained with Hoechst dye (1:1000) and the cells were observed using fluorescence microscopy (Leica DM IL LED).

### 2.10. Western Blot Analysis

T-84 cells were seeded into 6-well plates (3 × 10^5^ c/w) and exposed to the extracts for 24 h or 72 h. The cells were then detached, centrifuged and lysed with RIPA buffer (Thermo Fisher Scientific). Protein concentrations were quantified using the Bradford assay (Sigma-Aldrich). The Western blot protocol was adapted from Peña et al. (2023) [36]. In brief, proteins were denatured, separated by 10–12% SDS-PAGE and transferred to a nitrocellulose membrane. Protein-containing membranes were blocked with 5% (*w/v)* nonfat dry milk and incubated with the primary antibodies described in Table S1 overnight at 4 °C. Subsequently, the membranes were incubated for 1 h with HRP-conjugated secondary antibodies (Table S1) and revealed with Amersham™ ECL detection reagents (Cytiva, Little Chalfont, UK). Protein bands were observed in the LAS-400 mini luminescent image analyzer (Fujifilm, Tokyo, Japan) and they were quantified with Quantity One 4.6.8 (Bio-Rad, Hercules, CA, USA). β-actin protein, detected with an anti-β-actin peroxidase antibody, was used as an internal control.

### 2.11. Clonogenicity Assay

Following a 72 h treatment with the extracts at 25% and 75% inhibitory concentrations (IC_25_ and IC_75_), viable T-84 cells were seeded in 12-well plates (200 c/w). After 12 days, the cells were fixed with 10% TCA (20 min, 4 °C), and stained with 0.4% SRB (20 min). The plates were then scanned, and the images were used for colony quantification by ImageJ.

### 2.12. RT-PCR

T-84 cells were seeded in 6-well plates (1 × 10^5^ c/w) and exposed to a 72 h treatment with the extracts. The RT-PCR was carried out according to the method described by Mesas et al. (2021) [35], with some modifications. In brief, the cDNA obtained by reverse transcription of 4 μg of RNA (Superscript IV reverse transcriptase; Invitrogen) was amplified by RT-PCR with a mixture of TB-Green PremixExTaqII, ROX (Takara Bio Europe, Saint-Germain-en-Laye, France) and specific primers (Table S2) using a StepOnePlus™ system (Applied Biosystems, Thermo Fisher Scientific). StepOne software V2.0 (Applied Biosystems) was used to determine Ct values and gene expression was calculated with the 2^−ΔΔCt^ method using GAPDH as endogenous gene.

### 2.13. Wound Healing Assay

The wound healing assay was performed as described by Cabeza et al. (2021) [37]. In brief, extracts were applied to previously wounded T-84 cells at nontoxic concentrations under serum-free conditions for 72 h. Images that were acquired daily with an Olympus CKX-41 microscope (Olympus Corporation, Tokyo, Japan) were analyzed using the ImageJ MRI_Wound_Healing_Tool plugin.

### 2.14. Angiogenesis Study by CAM Assay

The chick chorioallantoic membrane (CAM) angiogenesis assay was performed as previously described by Peña et al. (2023) [36]. In brief, on the 7th day of incubation under the recommended conditions, the treatments (40 µL) were applied over the CAM of the fertilized eggs inside a sterilized plastic ring, which was accessible through a window that was opened on the 3rd day. After 72 h of treatment, images of the CAM-treated area captured with a Motic SMZ-171 stereomicroscope (Motic, Barcelona, Spain) were analyzed using the FIJI plugin “Vessel Analysis”. Ethical approval was not required as per EU guidelines. In the EU, the CAM assay is not considered an animal experiment by law, and therefore does not require ethical approval.

### 2.15. Antioxidant Activity In Vitro

HT-29 cells seeded in 96-well plates (2.5 × 10^4^ c/w) were used to evaluate the antioxidant activity of the extracts, based on the protocol described by Cabeza et al. (2021) [37]. In brief, HT-29 cells, previously treated with non-toxic concentrations of the extracts for 24 h, were exposed to oxidative stress with H_2_O_2_ for 6 h, under serum-free culture conditions, and cell viability was determined using the MTT (3-[4,5-dimethylthiazol-2-yl]-2,5 diphenyl tetrazolium bromide) assay.

### 2.16. Determination of Detoxifying Enzymes Induction Capacity

A methodology for determining detoxifying enzyme activity was described by Fuel et al. (2021) [38]. In brief, cytosolic fractions from HT-29 cells (1.5 × 10^6^ c/T25 flasks) treated with the extracts or sulforaphane (SFN; Sigma-Aldrich) for 48 h, were obtained by cell detachment, sonication and centrifugation (10,000× *g*, 5 min, 4 °C). The enzymatic activity of glutathione S-transferase (GST) (0.0096 μM^−1^ · cm^−1^) was measured via colorimetric increase at 340 nm induced by the reaction of the cytosolic fractions with glutathione and 1-chloro-2,4-dinitrobenzene (Sigma-Aldrich) in phosphate buffer. For NAD(P)H quinone oxidoreductase (QR) enzymatic activity (0.0205 μM^−1^ · cm^−1^), the colorimetric reduction of dichlorophenolindophenol (Sigma-Aldrich) was measured at 600 nm using a UV-Vis UV-1900i spectrophotometer (Shimadzu, Duisburg, Germany).

### 2.17. Bioguided Fractionation of Active Extracts

A fractionation process was conducted using Sep-Pak C18 Plus Short cartridges (Waters, Milford, MA, USA) following a modified version of the protocol described by Peña et al. (2023) [36]. In brief, the active seed extracts (10 mg/mL in 10 mM/20 mM Trizma/NaCl) were applied to preconditioned C18 cartridges, and five fractions were eluted with a gradient of acetonitrile (ACN, 20% to 100%) (Sigma-Aldrich). The fractions were lyophilized and resuspended in purified water for in vitro experiments and characterization. The cytotoxic potential of the fractions was tested on T-84 cells (2000 c/w in 96-well plates) using the MTT assay [36].

### 2.18. Chromatographic Studies by UPLC-MS Analysis

The active extracts were analyzed using UPLC Acquity I Class (Waters) coupled with TOF MS LCT Premier (Waters). The fractions were analyzed using a more precise and appropriate UPLC-MS equipment composed of UPLC Acquity H Class (Waters) coupled with QTOF MS Synap G2 (Waters). The conditions and parameters established for both analyses are specified in Table S3. The resulting data such as retention times (RT) and [M-H]^-^ *m*/*z* were analyzed with MassLynx V4.1 software (Waters), and a search for suggested molecular formulas in the Dictionary of Natural Products or ChemSpider databases led to the tentative identification of bioactive compounds.

### 2.19. Statistical Analysis

The results were expressed as the mean value ± SD of the replicates. Statistical significance (*p*-value < 0.05) was determined by Student’s t-test using the Statistical Package for the Social Sciences (SPSS) v28.0.1 software.

## 3. Results

### 3.1. Proliferation and Synergy Assay

**Figure 1:**
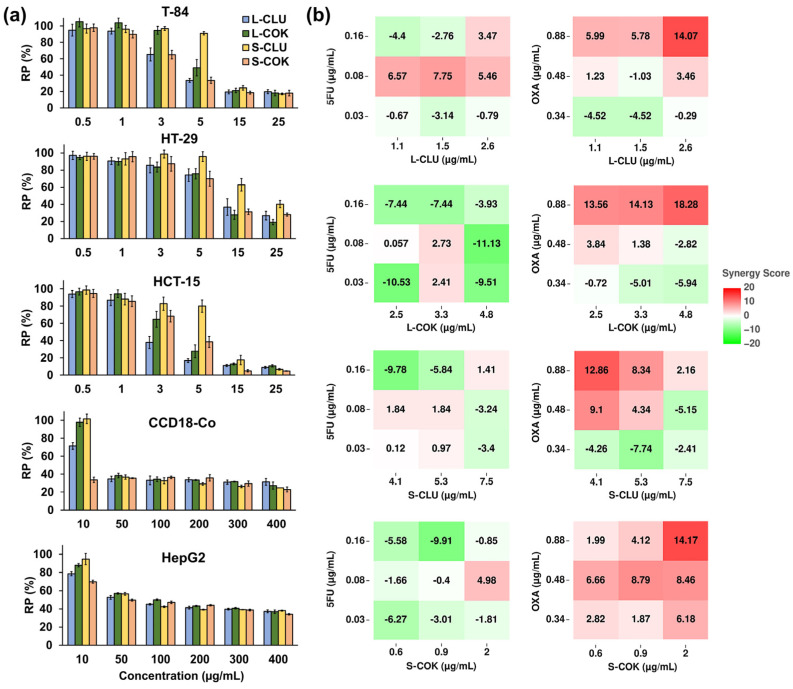
Antitumor potential of cEtOH from the leaves (L) and seeds (S) of CLU and COK plants alone or in combination with chemotherapeutic drugs. (**a**) Relative proliferation (RP, %) of cell lines treated with cEtOH for 72 h was determined by SRB assay. Data are the mean ± SD of three replicates from two different extractions. (**b**) HSA synergy scores of T-84 cells co-treated with cEtOH and 5-FU or OXA were plotted on heatmaps with red for synergy (>10), white for additive effect (from −10 to 10) and green for antagonism (<−10), using SynergyFinder Plus.

Four types of extracts, cold and reflux ethanolic extracts and protein extracts and hydrolysates, were obtained from eight leaf samples from Cucurbitaceae plants and tested on the T-84 CRC cell line (Table 1). Only the extracts from the leaves of two interspecific hybrids from the *Cucurbita* genus, CLU01002 (CLU) and COK01001 (COK), showed a significant cytotoxicity, highlighting that the cold ethanolic extract (cEtOH) treatment reached IC_50_ (half maximal inhibitory concentration) values of 3.90 and 8.84 µg/mL, respectively. Based on these results, cEtOH extracts from both the leaves (L) and seeds (S) of the CLU and COK plants were obtained and tested on different cell lines. As shown in Figure 1a, all of the extracts induced a significant dose-dependent decrease in cell viability, highlighting the low IC_50_ values of the cEtOH from the L-CLU sample in the T-84 and HCT-15 cells (3.90 and 2.51 µg/mL, respectively), from the L-COK sample in the HCT-15 cells (3.76 µg/mL) and from the S-COK sample in the T-84 cells (3.92 µg/mL) (Table 2). Interestingly, the highest IC_50_ values for most of the treatments were observed in the non-tumor cell line CCD-18Co and the hepatocarcinoma cell line HepG2, which were used as controls.

On the other hand, the combined treatment of the L-CLU and S-COK extracts with 5-FU and OXA showed promising results in the synergy HSA model analysis (Figure 1b). The highest HSA synergy score (18.28) was reached with the combination of OXA (0.88 µg/mL) and cEtOH from L-COK (4.8 µg/mL). However, a slight synergistic effect (>10 but <20) was observed with the combination of the highest dose of OXA (0.88 µg/mL) and the cEtOH extracts (2.6 µg/mL of L-CLU; 2.5, 3.3, and 4.8 µg/mL of L-COK; 4.1 µg/mL of S-CLU; and 2 µg/mL of S-COK). The combined treatment with 5-FU showed an additive effect (values between −10 and 10). On the other hand, the analysis using the Bliss, Loewe and ZIP models revealed that most of the combinations of extracts with 5-FU showed an antagonistic tendency (values < −10), whereas the effect of co-treatment with OXA was mostly additive (Figures S1–S3).

### 3.2. Cell Cycle and Apoptosis Assays

**Figure 2:**
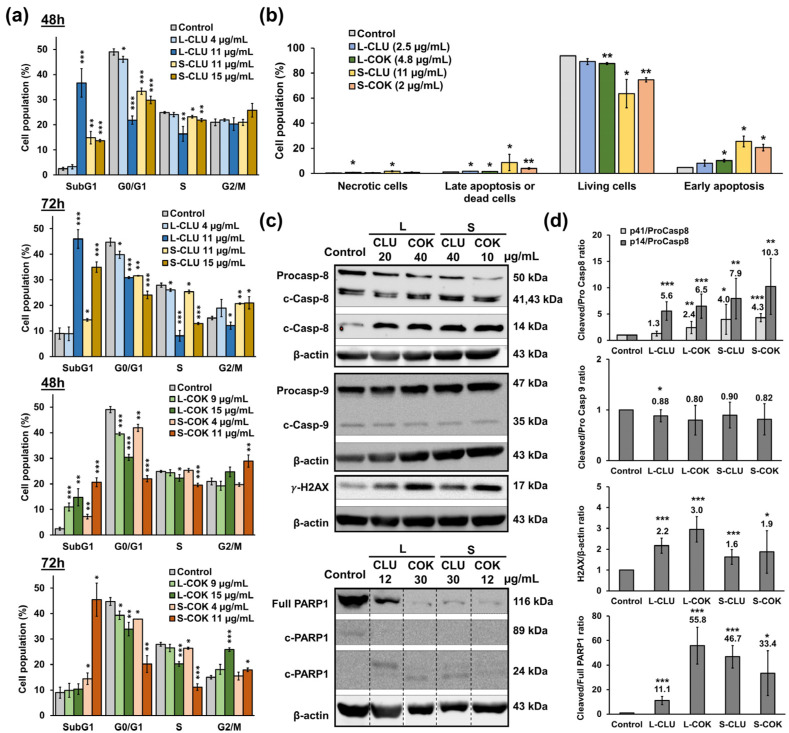
Cell cycle and apoptosis analysis of T-84 cells treated with cEtOH extracts from CLU and COK plants. (**a**) Cell population distribution of T-84 cells exposed to cEtOH from the leaves (L) and seeds (S) of CLU and COK for cell cycle analysis (48 and 72 h) and (**b**) for the study of apoptosis (48 h) using flow cytometry. Results are expressed as mean ± SD of three replicates. (**c**) Representative Western blot images of T-84 cells treated with cEtOH from L and S of CLU and COK for 24 h (caspase-8 and -9 and γ-H2AX) and 72 h (PARP1). WB images of PARP1 were obtained by splicing different lanes. (**d**) Relative protein expression was calculated as the mean ± SD of three measurements from three Western blot experiments. Statistical significance compared to control cells: *p* value < 0.05 (*); <0.01 (**); <0.001 (***).

The flow cytometry results showed a significant increase in the percentage of T-84 cells in the G_2_/M phase after treatment with the S-CLU (11 and 15 µg/mL), L-COK (15 µg/mL) and S-COK (11 µg/mL) extracts for 72 h (Figure 2a and Figure S4a). In addition, the highest concentrations of most of the extracts significantly increased the percentage of cells in the subG_1_ phase and decreased the proportion in the G_0_/G_1_ and S phases at 48 or 72 h. Moreover, a significant increase in T-84 early apoptotic cells treated with cEtOH from S-CLU (11 µg/mL) and L- and S-COK (4.8 and 2 µg/mL, respectively) was detected in comparison to non-treated cells (Figure 2b and Figure S4b). Fluorescent microscopy images corroborated the occurrence of apoptotic death in the treated cells (Figure S5). A significant increase in the cleaved/procaspase-8 ratio in the treated T-84 cells was demonstrated by Western blot (Figure 2c,d). It is worth highlighting the increase in cleaved caspase-8 (14 kDa) after the use of the L- and S-CLU extracts (5.6 and 7.9-fold, respectively) and the L- and S-COK extracts (6.5 and 10.3-fold, respectively). By contrast, only a slight but significant decrease in the cleaved/procaspase-9 ratio was observed after L-CLU extract treatment. Furthermore, the cleaved/full-length PARP1 ratio was significantly increased after the cells were treated with L-CLU (11.1-fold), S-CLU (46.7-fold), L-COK (55.8-fold), and S-COK (33.4-fold) extracts, indicating a decrease in the expression of full-length PARP1. Finally, a significant increase in γ-H2AX expression was detected after the use of L- and S-CLU extracts (2.2- and 1.6-fold, respectively) and L- and S-COK (3- and 1.9-fold, respectively) compared to the control cells.

### 3.3. Autophagy and Immunofluorescence Assays

**Figure 3:**
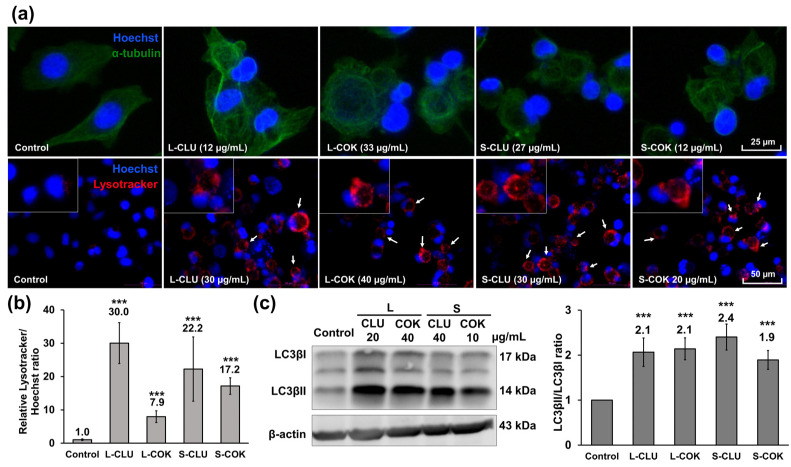
Cellular microtubule organization and autophagy process in T-84 cells treated with cEtOH extracts from CLU and COK plants. (**a**) Representative fluorescent microscopy images of α-tubulin (upper-green, amplified from 20× magnification) and acidic vesicles (lower-red, indicated by arrows, original 40× magnification) in cells treated with cEtOH from the leaves (L) and seeds (S) of CLU and COK for 24 h. Cell nuclei (blue) were stained with Hoechst 33342. (**b**) Lysotracker/Hoechst staining ratio relative to control cells was quantified using ImageJ software as the mean ± SD of three images from four replicates. (**c**) Determination of LC3B protein expression in T-84 cells treated with cEtOH from L and S of CLU and COK (24 h) by Western blot. The LC3B-II/LC3B-I ratio was calculated as the mean ± SD of three Western blot replicates. Statistical significance compared to control cells: *p* value < 0.001 (***).

Fluorescence microscopy showed the presence of multinucleated cells, disorganized and aggregated microtubules and intercellular bridges in the T-84 cells treated with cEtOH from CLU and COK (Figure 3a and Figure S6). Moreover, the presence of autophagy vesicles was detected through staining with Lysotracker (Figure 3a and Figure S7). The Lysotracker/Hoechst staining ratio was strongly increased in the cells treated with cEtOH from L and S of CLU (30 and 22.2-fold, respectively) and COK (7.9 and 17.2-fold, respectively) compared to the control cells (Figure 3b). These cells also showed a significant increase in the LC3B-II autophagy biomarker and the LC3B-II/LC3B-I ratio (≈2-fold) (Figure 3c).

### 3.4. Clonogenicity, Migration and Angiogenesis Assays

**Figure 4:**
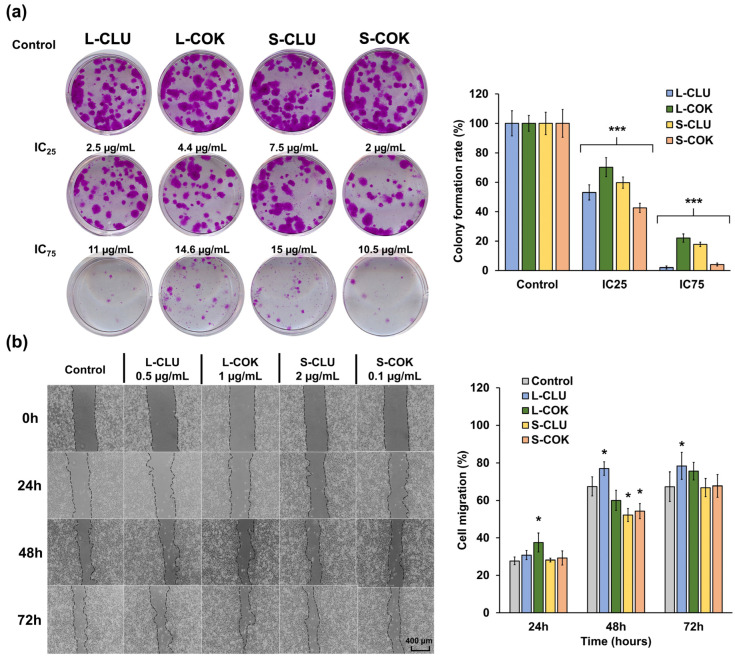
Study of clonogenicity and cell migration of T-84 cells treated with cEtOH extracts from CLU and COK plants. (**a**) Representative images of T-84 cells colonies (12 days) stained with SRB and graph representation of the percentage of colony formation. Cells were pretreated (72 h) with cEtOH (IC_25_ and IC_75_) from the leaves (L) and seeds (S) of CLU and COK. (**b**) Representative light microscopy images of wound healing assay in T-84 cells treated with cEtOH from L and S of CLU and COK (4× magnification), and graphical representation of the percentage of cell migration. Data were represented as the mean ± SD of triplicate cultures. Statistical significance compared to control cells: *p* value < 0.05 (*); <0.001 (***).

**Figure 5:**
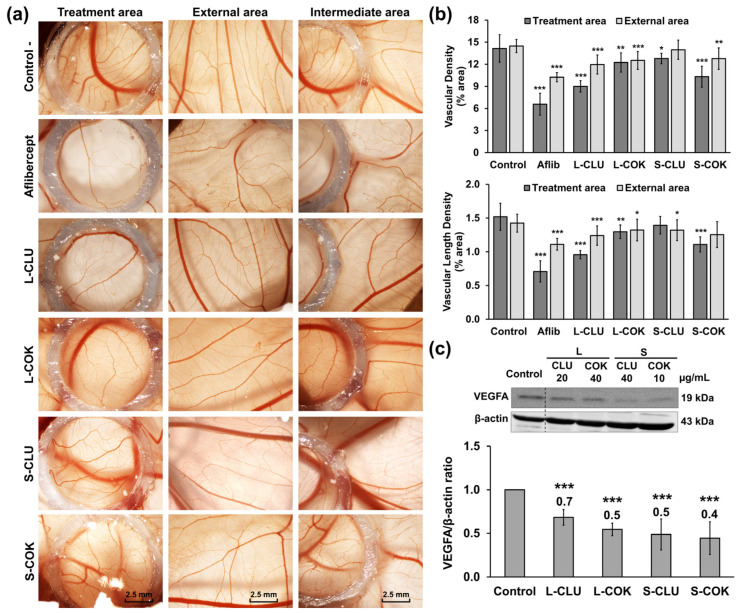
Study of the antiangiogenic potential of cEtOH extracts from CLU and COK plants. (**a**) Stereomicroscope images of the CAM (treatment, external and intermediate areas respect to the ring) treated with saline solution (40 μL/egg; negative control), aflibercept (4 mg/mL; positive control) and cEtOH from the leaves (L) and seeds (S) of CLU and COK (250 μg/mL) for 72 h (3× magnification). (**b**) Vascular density and vascular length density areas presented as the mean ± SD (n = 4). (**c**) Western blot of T-84 cells’ VEGFA expression after treatment (24 h) with cEtOH from L and S of CLU and COK. WB images of VEGFA were obtained by splicing different lanes. The VEGFA/β-actin ratio was presented as the mean ± SD of three measures from three Western blot experiments. Statistical significance compared to negative control: *p* value < 0.05 (*); <0.01 (**); <0.001 (***).

All of the cEtOH extracts showed a concentration-dependent inhibition of the T-84 cells’ clonogenicity (Figure 4a). Concrete evidence shows that a pretreatment with cEtOH from L and S of CLU and COK (IC_75_) strongly reduced the colony formation rates (2.1%, 17.8%, 22.1% and 4.1%, respectively) in the T-84 cells. By contrast, only the cEtOH from S-CLU and S-COK significantly decreased the cells’ migratory capacity (15.25% and 13.21%, respectively) at 48 h (Figure 4b). On the other hand, the antiangiogenic activity of the extracts was evaluated by the CAM assay in chick embryos. As shown in Figure 5a,b, a significant reduction in vascular density areas (5.15 and 3.84%) and vascular length density areas (0.56 and 0.41%) was detected with the L-CLU and S-COK extract treatments, respectively. These results were comparable to those for aflibercept (positive control), which induced decreases of 7.58% and 0.81% in vascular density and vascular length density areas, respectively. Finally, a significant reduction in VEGFA expression was observed in the Western blot analysis after all extract treatments in comparison to the control cells (Figure 5c).

### 3.5. Expression of CSC Genes by Real Time PCR

Treatment with most of the extracts induced a significant decrease in the relative expression of the cancer stem cell (CSC)-related genes CD24, CD133, NANOG and OCT-4. The L-CLU extract induced the greatest reductions in CD24, OCT-4 and SOX-2 expression (0.77 ± 0.02, 0.54 ± 0.1 and 0.14 ± 0.01, respectively), and the L-COK extract led to the greatest reductions in CD133 and NANOG (0.42 ± 0.02 and 0.49 ± 0.06, respectively). However, a significant increase in the expression of CD44 and SOX2 (1.38 ± 0.21; and 1.44 ± 0.27, respectively) was detected, except for the cEtOH from L-CLU (Figure S8).

### 3.6. In Vitro Antioxidant and Chemopreventive Activity

**Figure 6:**
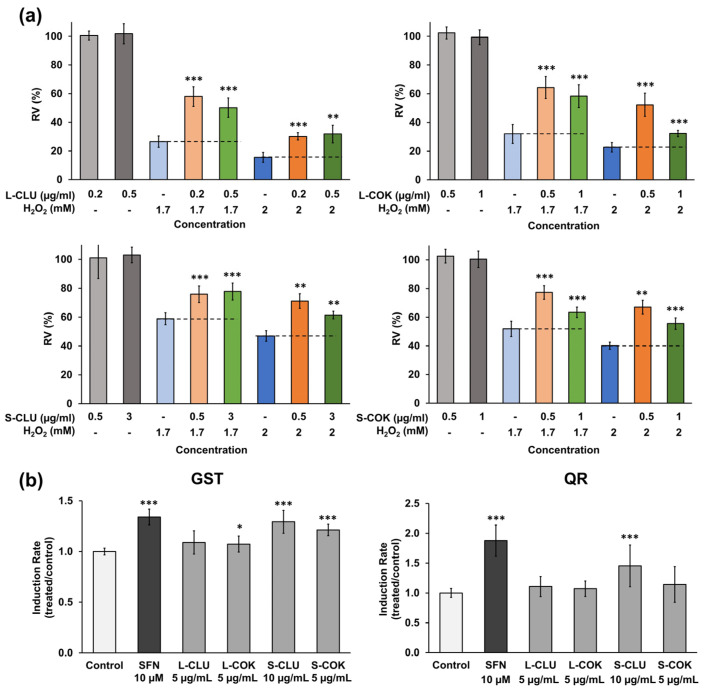
In vitro antioxidant and chemopreventive activity of cEtOH extracts from CLU and COK plants. (**a**) Percentage of relative viability of cells pretreated with cEtOH extracts (non-toxic doses) and exposed to hydrogen peroxide (H_2_O_2_). Relative viability (RV, %) was calculated by MTT assay and results are expressed as the mean ± SD of 8 replicates. The statistically significant differences were calculated compared to treated cells treated with H_2_O_2_: *p* value < 0.01 (**); <0.001 (***). (**b**) Glutathione S-Transferase (GST) and NAD(P)H quinone oxidoreductase (QR) activity in cytosolic fractions obtained from HT-29 cells treated with cEtOH. Sulforaphane (SFN) was used as positive control. Statistical significance compared to control cells: *p* value < 0.05 (*); <0.001 (***).

The results obtained showed a protective effect of the extracts on the viability of H_2_O_2_-exposed cells (Figure 6a). The most relevant results were an increase in cell proliferation up to 31.62% and 32.39% (0.2 µg/mL L-CLU and 0.5 µg/mL L-COK extracts, respectively, both with 1.7 mM H_2_O_2_) and up to 24.18% and 26.93% (0.5 µg/mL S-CLU and 0.5 µg/mL S-COK extracts, respectively, both with 2 mM H_2_O_2_). Moreover, the chemopreventive activity of the extracts was shown by a significant increase in the GST enzyme induction rate (IR) compared to the negative controls (IR: 1.29 ± 0.11 for S-CLU and 1.21 ± 0.06 for S-COK extracts) (Figure 6b). However, the activity of the QR enzyme was only increased by the S-CLU extract (IR: 1.45 ± 0.35).

### 3.7. Analysis of the Bioactive Composition by UPLC-MS

**Figure 7:**
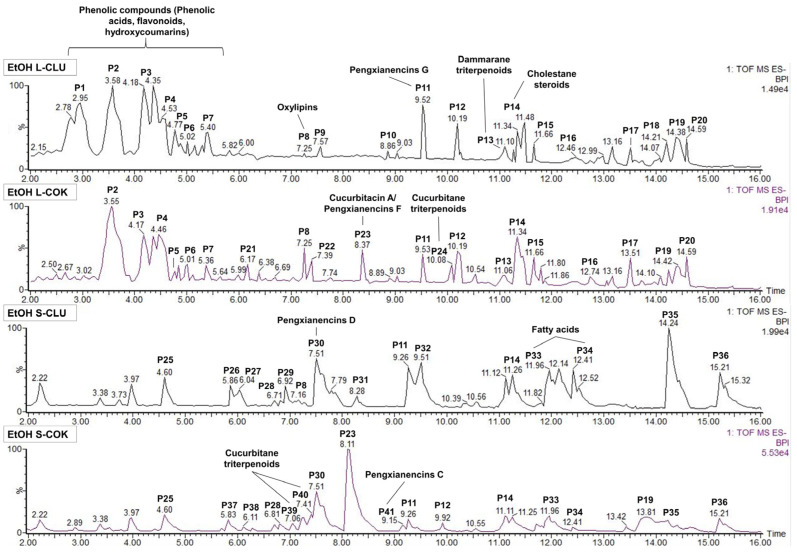
UPLC-MS chromatographic profiles of cEtOH obtained from leaves and seeds of CLU and COK plants. The highest peaks were highlighted and tentatively identified in mass spectrometry databases such as the Dictionary of Natural Products and the Chemspider database.

Several bioactive compounds have been tentatively identified in the chromatographic profiles of cEtOH from the leaves and seeds of CLU and COK by UPLC-MS (Figure 7). Leaf extracts were mainly composed of phenolic compounds such as phenolic acids, flavonoids and hydroxycoumarins, and of dammarane and cucurbitane-type triterpenoids, whereas in the seed extracts, curcurbitane triterpenoids and fatty acids were predominant (Table S4). One of the largest peaks in the four extracts, P11 (RT: 9.2–9.5 min) with *m*/*z* of 617.33 has been tentatively associated with the cucurbitane-type triterpenoid pengxianencins G (C_34_H_50_O_10_). Furthermore, the highest peak in S-COK extract, P23 (RT: 8.1–8.3 min) with an *m*/*z* of 573.31, identified as cucurbitacin A or pengxianencins F (C_32_H_46_O_9_), was exclusively detected in the COK-derived extracts. Similarities were also detected in the composition of seed extracts, including P30 (RT: 7.5 min) with an *m*/*z* of 575.32 which was identified as pengxianencins D (C_32_H_48_O_9_). Finally, the presence of other cucurbitane-type triterpenoids such as balsaminoside B (C_36_H_60_O_8_ in P24) in the L-COK extract, or colocynthenin F (C_32_H_48_O_10_ in P40) and pengxianencins C (C_34_H_52_O_10_ in P41) in the S-COK extract has been suggested.

### 3.8. Bioassay-Guided Fractionation of Seed Extracts

The fractions (40%, 60% and 80%) derived from both the CLU and COK seed extracts significantly decreased cell viability, reaching IC_50_ values lower than 10 µg/mL for samples such as the 60% ACN fraction (Figure S9). Th e UPLC-MS analysis showed that most of the peaks detected in the whole extracts (RT: 12–16 min) were present in the 40%, 60% or 80% fractions, but not in the 20% or 100% ones (Figures S10 and S11). The highest peaks in the 40% and 60% S-CLU fractions (Table S5, Figure S10) were identified as pengxianencins C (P9), D (P5), E (P6), G (P8); cucurbitacin R, F or O (P6), C, Q; or dihydrocucurbitacin B (P9). The analysis of the S-COK fractions (Table S6, Figure S11) suggested the presence of cucurbitacins A (P8), H or G (P4), K or J (P5), D or L (P9); pengxianencins D (P6), F (P8), G (P10); and colocynthenin F (P7) in the complete S-COK extracts and its respective 40%, 60% and 80% fractions.

## 4. Discussion

CRC is a major healthcare problem, and the low specificity of cytotoxics used in the treatment and/or the development of resistance to these drugs is becoming an increasing concern. The present study evaluates the bioactive potential of the extracts from Cucurbitaceae plants for their application in the prevention and treatment of CRC.

Previous studies demonstrated that extracts or compounds isolated from cucurbit plants, such as lectin from fruit exudates of *Praecitrullus fistulosus*, exhibited significant antitumor activity against CRC cell lines (HT-29 and HCT-116) [39]. In our study, the extracts from two of the eight cucurbit varieties tested (CLU and COK) induced a high cytotoxicity in three CRC cell lines (T-84, HCT-15 and HT-29) (IC_50_ < 20 µg/mL). Interestingly, one of these cell lines, HCT-15, was characterized by a resistance phenotype [40]. Our results suggest that their antitumor activity may be specific and non-hepatotoxic, since the extracts showed a lowest cytotoxicity against a non-tumor colon cell line (CCD-18Co), and hepatocarcinoma cells (HepG2) are usually used to evaluate hepatotoxicity [41]. Recently, a fruit extract from *Diplocyclos palmatus* L. [42] and a crude acetone extract of *Momordica balsamina* leaves [43] demonstrated antitumor activity against HT-29 CRC cells but with a higher LC_50_ and IC_50_ (46.88 µg/mL and >200 µg/mL, respectively) than those found in our extracts (10–20 µg/mL). Furthermore, a slight synergism with the use of cEtOH/OXA was demonstrated, whereas the association with 5-FU only showed an additive effect. This fact may be clinically relevant and may support other similar synergisms, such as those between cucurbitacin E/5-FU and OXA [44], or *Momordica charantia* bitter melon extract/doxorubicin in CRC cells [45].

Cucurbitacins can disrupt the cytoskeleton and induce apoptosis and cell cycle arrest in tumor cells [12,21,46]. CLU and COK extracts also induced a remarkable increase in G_2_/M phase and early apoptosis, as suggested by flow cytometry and morphological changes. In fact, apoptosis by CLU and COK extracts could be mediated by the caspase-dependent extrinsic pathway, since an overexpression of the active fragment of caspase-8 (14 kDa) was detected. However, the expression of caspase-9 was similar to that of untreated cells, suggesting that the intrinsic pathway may not be involved. Furthermore, increased expression of PARP1 inactive fragments (24 kDa), which facilitates caspase-mediated DNA fragmentation during apoptosis [47], was observed. Finally, γ-H2AX, which appears after the introduction of double-strand breaks in DNA during apoptosis [48], was also overexpressed in T-84 cells after treatment with the extracts. On the other hand, some cucurbit-derived compounds, such as cucurbitacin B and E, can induce autophagy in tumor cells [49,50]. During autophagosome formation, the soluble form of LC3B (LC3B-I) is transformed into a vesicular form (LC3B-II) that has been used as an autophagy biomarker [51]. Interestingly, our results showed an increased number of lysosomes and a higher LC3B-II/LC3B-I expression ratio in cells treated with cEtOH from CLU and COK, suggesting the activation of an autophagy process.

Our results demonstrate that both CLU and COK cEtOH not only decreased the proliferation capacity of CRC cells, but were also able to significantly inhibit the clonogenicity and, to a lesser extent, the migration capacity of tumor cells. However, other compounds such as cucurbitacin C and I have shown a higher inhibition of the migration of CRC cell lines [52,53]. Furthermore, the significant decrease in vascular areas in the CAM of chicken embryos, and the reduced expression of the proangiogenic protein VEGFA [54] in T-84 cells, suggested a negative effect on vessel formation, which may be clinically relevant since angiogenesis is closely related to metastatic capacity [55]. Indeed, previously, Kim and Kim (2015) [56] and Piao et al. (2018) [57] demonstrated a similar property when cucurbitacin B and I were tested. cEtOH from CLU and COK were also able to decrease the expression of genes associated with the CRC CSC phenotype such as CD24, CD133, NANOG and OCT-4, which have been associated with drug resistance, recurrence and metastasis [58,59]. On the other hand, S-CLU extract was able to positively modulate the activity of GST and QR enzymes, which was correlated with an antioxidant effect and a chemoprotective capacity against carcinogens, oxidants and other toxic chemicals, which have been shown to be the key factor in cancer prevention [60]. In fact, treatment with non-toxic concentrations of cEtOH extracts demonstrated a high protection against H_2_O_2_-induced oxidative damage in HT-29 cells. These antioxidant and chemopreventive properties have been widely described in other cucurbits previously [4,61].

Finally, the antitumor activity of CLU and COK extracts could be attributed to the presence of cytotoxic cucurbitane-type triterpenoids such as pengxianencins (C, D, E, F or G) [62] and cucurbitacins (A, C, D, F, G, H, J, K, L, O, Q or R) [63,64] which have been tentatively identified by UPLC-MS. In particular, we highlight the presence of pengxyanencins G in the four extracts and their respective active fractions, and of cucurbitacin A or pengxianencins F in the COK-derived extracts. In addition, phenolic compounds characterized by potent antioxidant, anti-inflammatory and antitumor properties [65], were also identified in the leaf extracts. Further research is needed to precisely identify and isolate the active compounds and to evaluate their preventive and therapeutic efficacy in in vivo CRC models.

## 5. Conclusions

The present work confirmed the importance of Cucurbitaceae plants as a source of extracts and compounds with biological activities applicable to CRC treatment and prevention. The cEtOH obtained from the leaves and seeds of two interspecific hybrids from the *Cucurbita* genus (CLU and COK) have demonstrated a potent antiproliferative effect on several CRC cell lines, and synergism with OXA. Several mechanisms including cytokinesis inhibition with G_2_/M arrest, an extrinsic apoptotic pathway, and autophagy may be involved. The extracts may also reduce tumor invasiveness and malignancy by decreasing the clonogenic and migratory capacity of tumors and downregulating the expression of specific CSCs genes in tumor cells as well as inhibiting angiogenesis. Moreover, the antioxidant and chemopreventive activities demonstrated by the extracts could be used as a prevention strategy for CRC. A comprehensive list of potential compounds in the active extracts has been proposed, including phenolic compounds, cucurbitane-type triterpenoids (cucurbitacins and pengxianencins) and fatty acids. However, additional research is necessary to confirm their presence and elucidate their role in the observed biological activities.

## Figures and Tables

**Table 1:** IC_50_ values (µg/mL) of extracts from leaves of Cucurbitaceae plants in T-84 CRC cell line.

Genus	Specie	Sample Code	cEtOH	rEtOH	PE	PH
*Cucurbita*	*C. pepo*	151SQ	784.2 ± 128.9	>1000	>1000	224.9 ± 49.3
		MURCIA	731.1 ± 10.5	>1000	>1000	388.6 ± 6.3
	*C. moshata*	BTN18010	>1000	>1000	>1000	367.5 ± 4.8
	*C. maxima*	ZP180235	>1000	>1000	>1000	372.1 ± 2.2
		ZP180301	>1000	>1000	534.7 ± 108.7	472.6 ± 67.4
	*Interspecific*	CLU01002	3.9 ± 0.3	5.5 ± 1.0	8.4 ± 0.7	42.1 ± 13.9
		COK01001	8.8 ± 1.2	10.4 ± 1.4	9.8 ± 0.3	30.4 ± 0.9
*Sechium*	*S. edule*	CHAYOTE	>1000	>1000	512.7 ± 101.6	251.0 ± 29.2

cEtOH (cold ethanolic extract); PE (protein extract); PH (protein hydrolysate); rEtOH (reflux ethanolic extract).

**Table 2:** IC_50_ values (µg/mL) of cEtOH from leaves and seeds of CLU and COK in tumor and non-tumor cell lines.

Sample	Plant Part	T-84	HCT-15	HT-29	CCD-18Co	HepG2
CLU	Leaves	3.90 ± 0.30	2.51 ± 0.27	11.36 ± 2.21	33.33 ± 0.53	66.11 ± 2.66
	Seeds	11.10 ± 0.31	9.71 ± 0.98	20.37 ± 2.29	41.86 ± 1.36	72.65 ± 3.50
COK	Leaves	8.84 ± 1.18	3.76 ± 0.44	10.33 ± 1.08	42.20 ± 2.04	99.12 ± 5.74
	Seeds	3.92 ± 0.28	4.18 ± 0.44	10.29 ± 1.35	<10	51.0 ± 5.10

CLU (CLU01002); COK (COK01001).

## Data Availability

Data available on request due to restrictions.

## Supplementary Materials

The following supporting information can be downloaded at: https://www.mdpi.com/article/10.3390/nu16234233/s1, Figure S1: Evaluation of synergistic effect of cEtOH extracts from CLU and COK plants with chemotherapy drugs by Bliss model; Figure S2: Evaluation of synergistic effect of cEtOH extracts from CLU and COK plants with chemotherapy drugs by Loewe model; Figure S3: Evaluation of synergistic effect of cEtOH extracts from CLU and COK plants with chemotherapy drugs by ZIP model; Figure S4: Cell cycle and apoptosis flow cytometry charts; Figure S5: Fluorescence images of T84 cells nuclei after treatment with cEtOH extracts from CLU and COK plants; Figure S6: Immunofluorescence stain of α-tubulin in T84 cells treated with cEtOH from CLU and COK plants; Figure S7: Fluorescence detection of autophagic vesicles in T84 cells treated with cEtOH from CLU and COK plants; Figure S8: CSC-related genes relative expression in T-84 treated with cEtOH extracts from CLU and COK plants; Figure S9: Percentage of relative proliferation of T-84 cells treated with fractions from CLU and COK seeds extracts; Figure S10: UPLC-MS chromatographic profiles of chromatographic fractions obtained from cEtOH of S-CLU; Figure S11: UPLC-MS chromatographic profiles of chromatographic fractions obtained from cEtOH of S-COK; Table S1: Primary and secondary antibodies used in the Western blot experiments; Table S2: Primers used in the qPCR experiments; Table S3: Experimental parameters of the two different UPLC-MS analysis; Table S4: UPLC-MS peak tentative identification in cEtOH obtained from leaves and seeds of CLU and COK; Table S5: UPLC-MS peak tentative identification in fractions obtained from cEtOH of S-CLU; Table S6: UPLC-MS peak tentative identification in fractions obtained from cEtOH of S-COK.
