# Supplementary material for: Evaluation of the Leaves and Seeds of Cucurbitaceae Plants as a New Source of Bioactive Compounds for Colorectal Cancer Prevention and Treatment
Source: Nutrients. 2024 Dec 7;16(23):4233. doi: 10.3390/nu16234233 (PMC11644257; doi:10.3390/nu16234233)
Supplement: Supplementary file 1 [file nutrients-16-04233-s001.zip › nutrients-3331888-supplementary.pdf]

# Evaluation of the Leaves and Seeds of Cucurbitaceae Plants as a New Source of Bioactive Compounds for Colorectal Cancer Prevention and Treatment

Mercedes Peña [1,2], Ana Guzmán [3], Cristina Mesas [1,2], Jesús M. Porres [4], Rosario Martínez [4], Francisco Bermúdez [3], Consolación Melguizo [1,2,5,*], Laura Cabeza [1,2,5,*] and Jose Prados [1,2,5]

## Supplementary material:

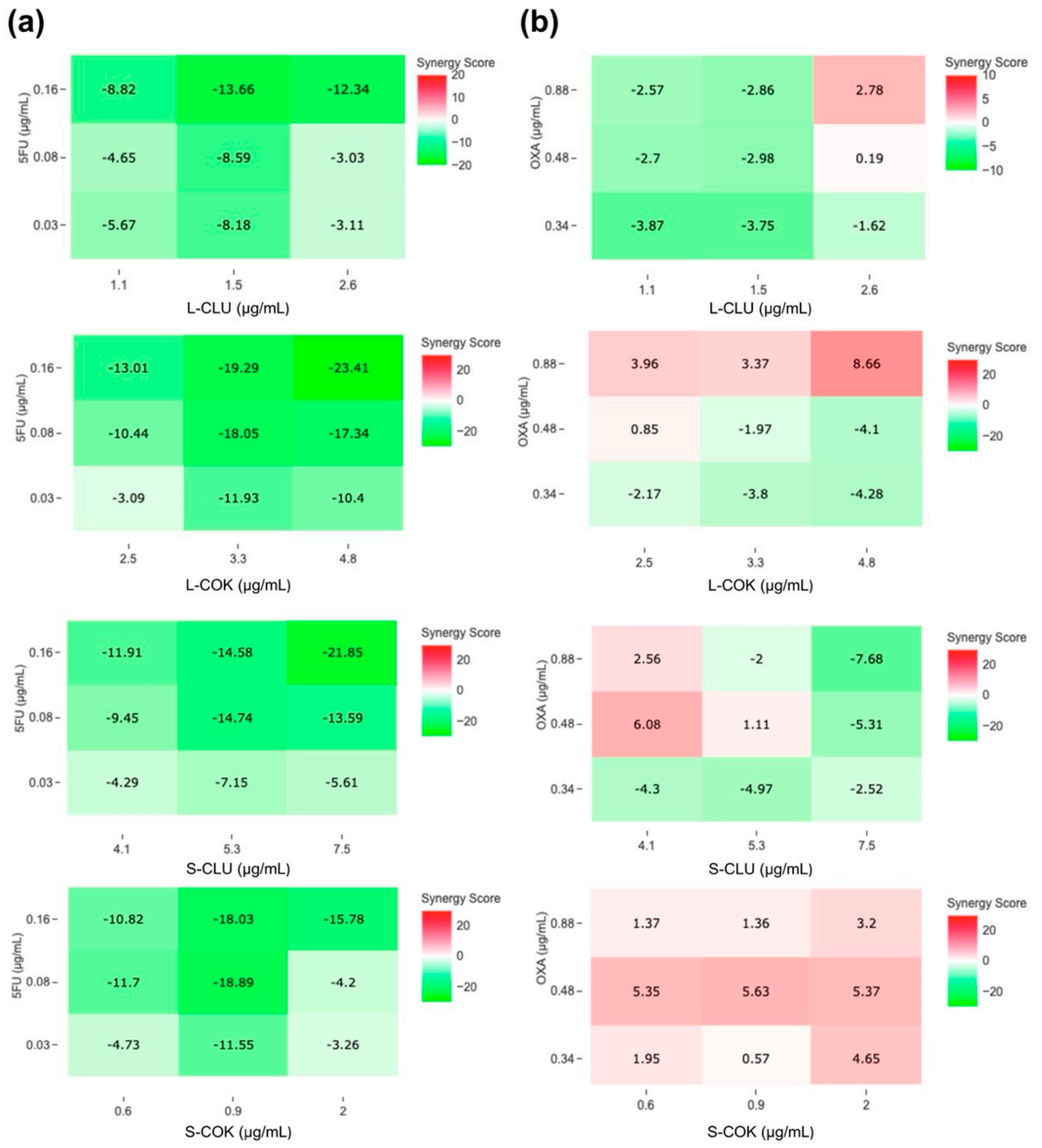


**Figure S1.** Evaluation of synergistic effect of cEtOH extracts from CLU and COK plants with chemotherapy drugs by Bliss model. T84 cells were treated with cEtOH extracts from leaves and

seed of CLU and COK plants in combination with **(a)** 5-FU or **(b)** OXA. Synergy scores calculated by the Bliss model were plotted on heat maps using SynergyFinder Plus, distinguishing areas of synergy in red (values >10), addition in white (from -10 to 10), or antagonism in green (<-10).

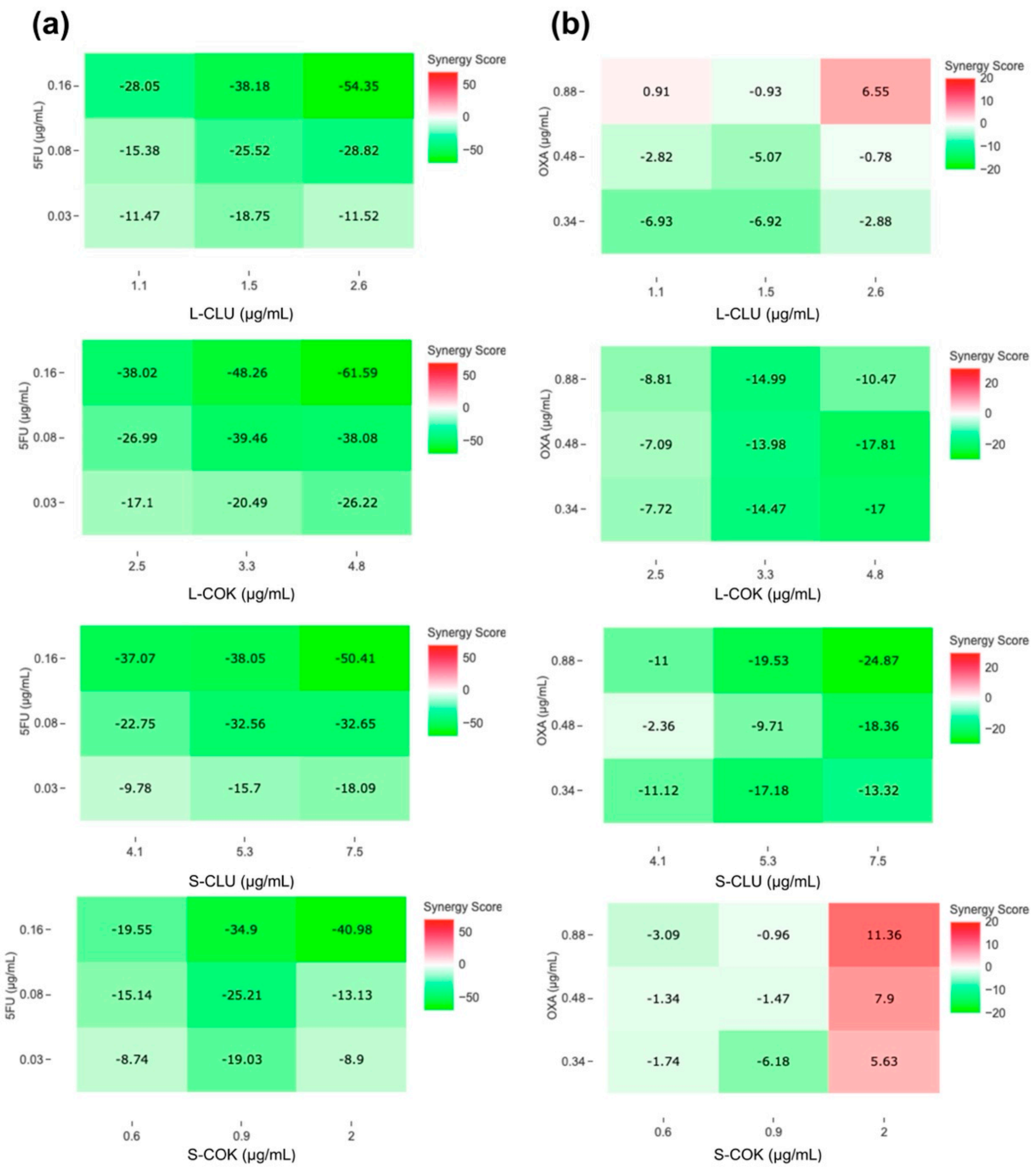


**Figure S2.** Evaluation of synergistic effect of cEtOH extracts from CLU and COK plants with chemotherapy drugs by Loewe model. T84 cells were treated with cEtOH extracts from leaves and seed of CLU and COK plants in combination with **(a)** 5-FU or **(b)** OXA. Synergy scores calculated by the Loewe model were plotted on heat maps using SynergyFinder Plus, distinguishing areas of synergy in red (values >10), addition in white (from -10 to 10), or antagonism in green (<-10).

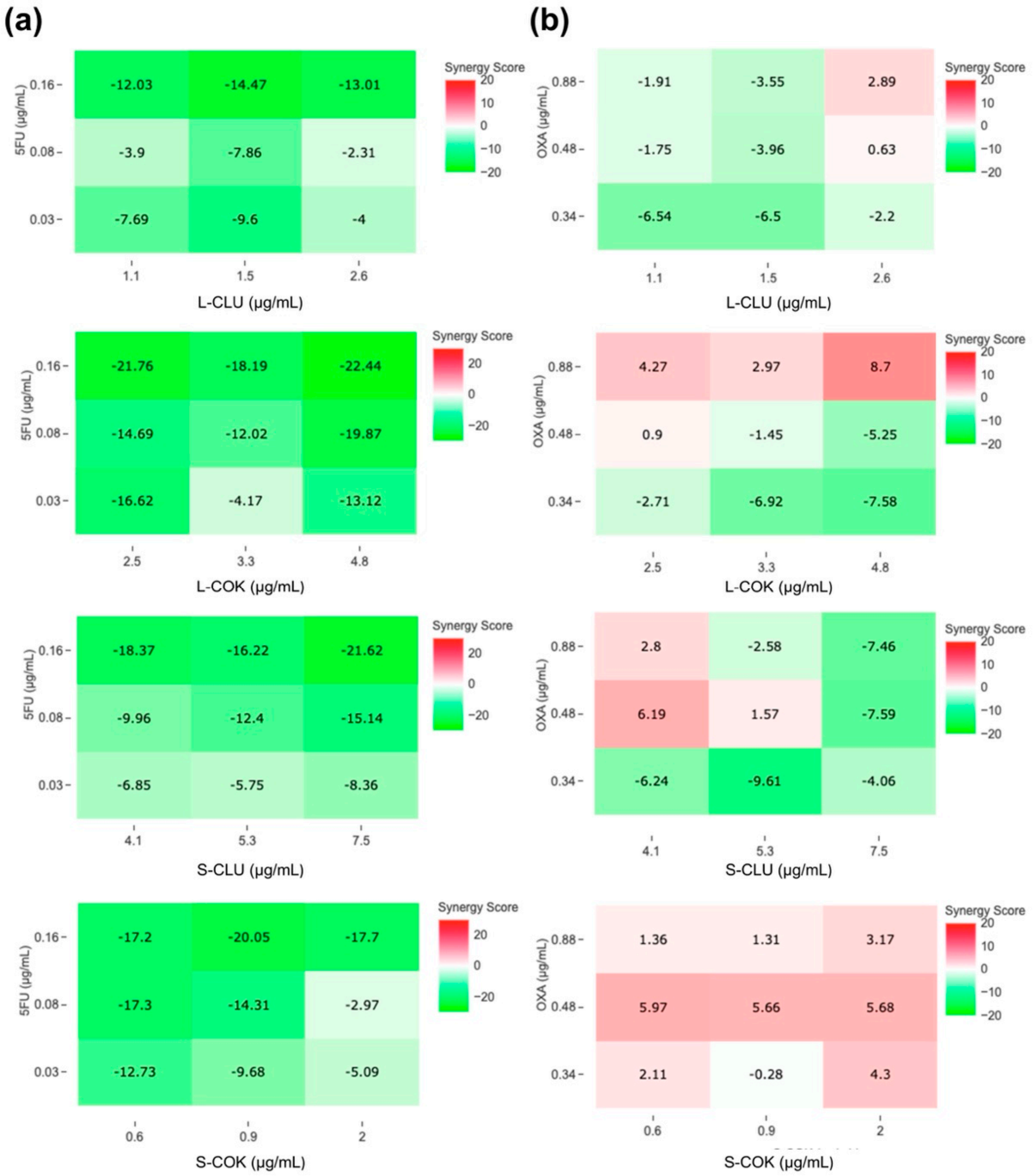


**Figure S3.** Evaluation of synergistic effect of cEtOH extracts from CLU and COK plants with chemotherapy drugs by ZIP model. T84 cells were treated with cEtOH extracts from leaves and seed of CLU and COK plants in combination with **(a)** 5-FU or **(b)** OXA. Synergy scores calculated by the ZIP model were plotted on heat maps using SynergyFinder Plus, distinguishing areas of synergy in red (values >10), addition in white (from -10 to 10), or antagonism in green (<-10).

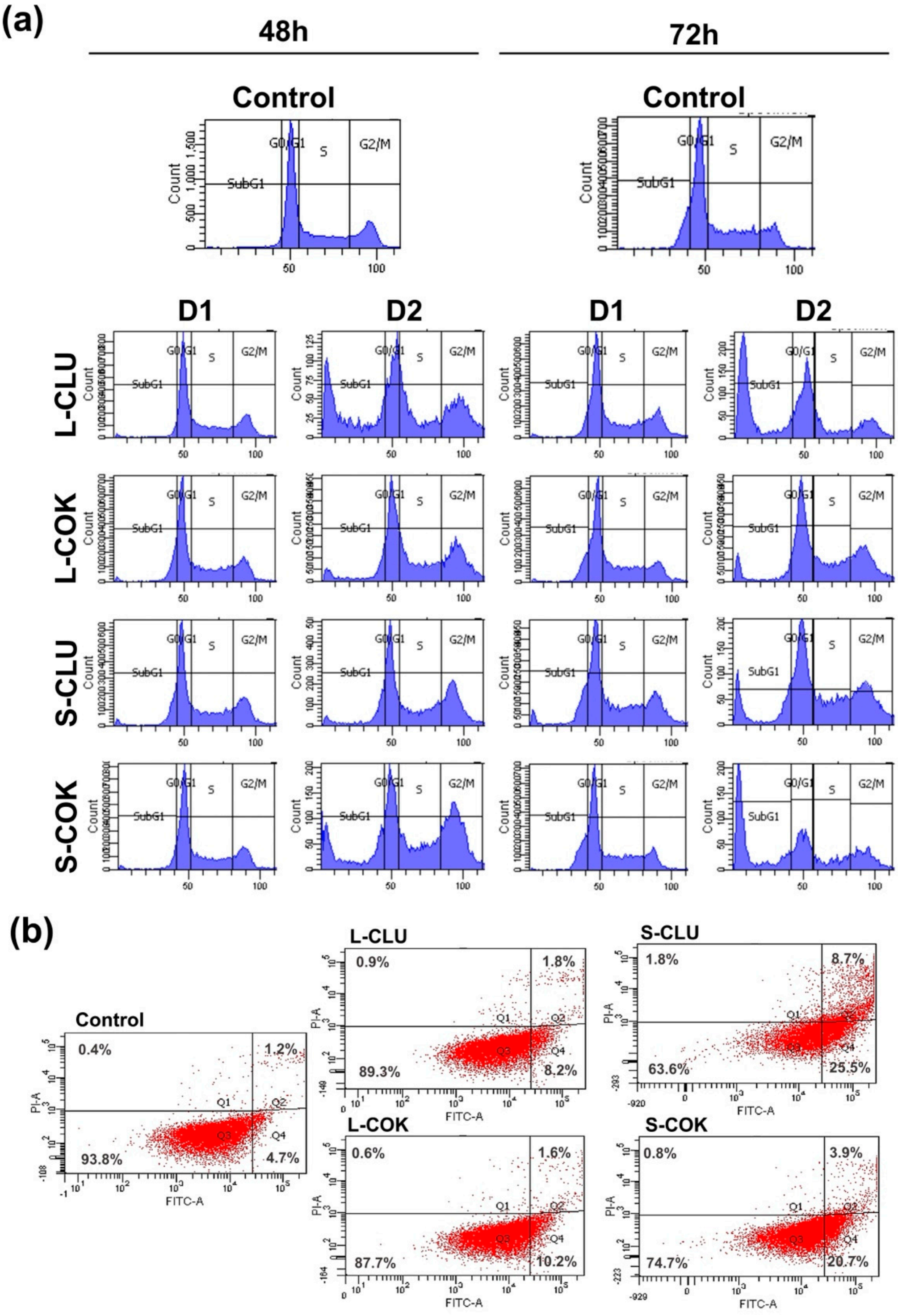


**Figure S4**. Cell cycle and apoptosis flow cytometry charts. **(a)** T84 cells were treated with two concentrations (D1 and D2) of cEtOH from L-CLU (4 and 11 µg/mL) and S-CLU (11 and 15 µg/mL), L-COK (9 and 15 µg/mL) and S-COK (4 or 11 µg/mL) for 48 and 72 hours for cell cycle study. **(b)** For apoptosis assay, T84 cells were treated with cEtOH from leaves and seeds of CLU (2.5 and 11 µg/mL, respectively) and COK (4.8 and 2 µg/mL, respectively) for 48 hours. Results are represented as dot plots with cell population distribution according to Annexin V-FITC and PI detection: necrotic (Q1), late apoptotic or dead (Q2), live (Q3) or early apoptotic (Q4) cells.

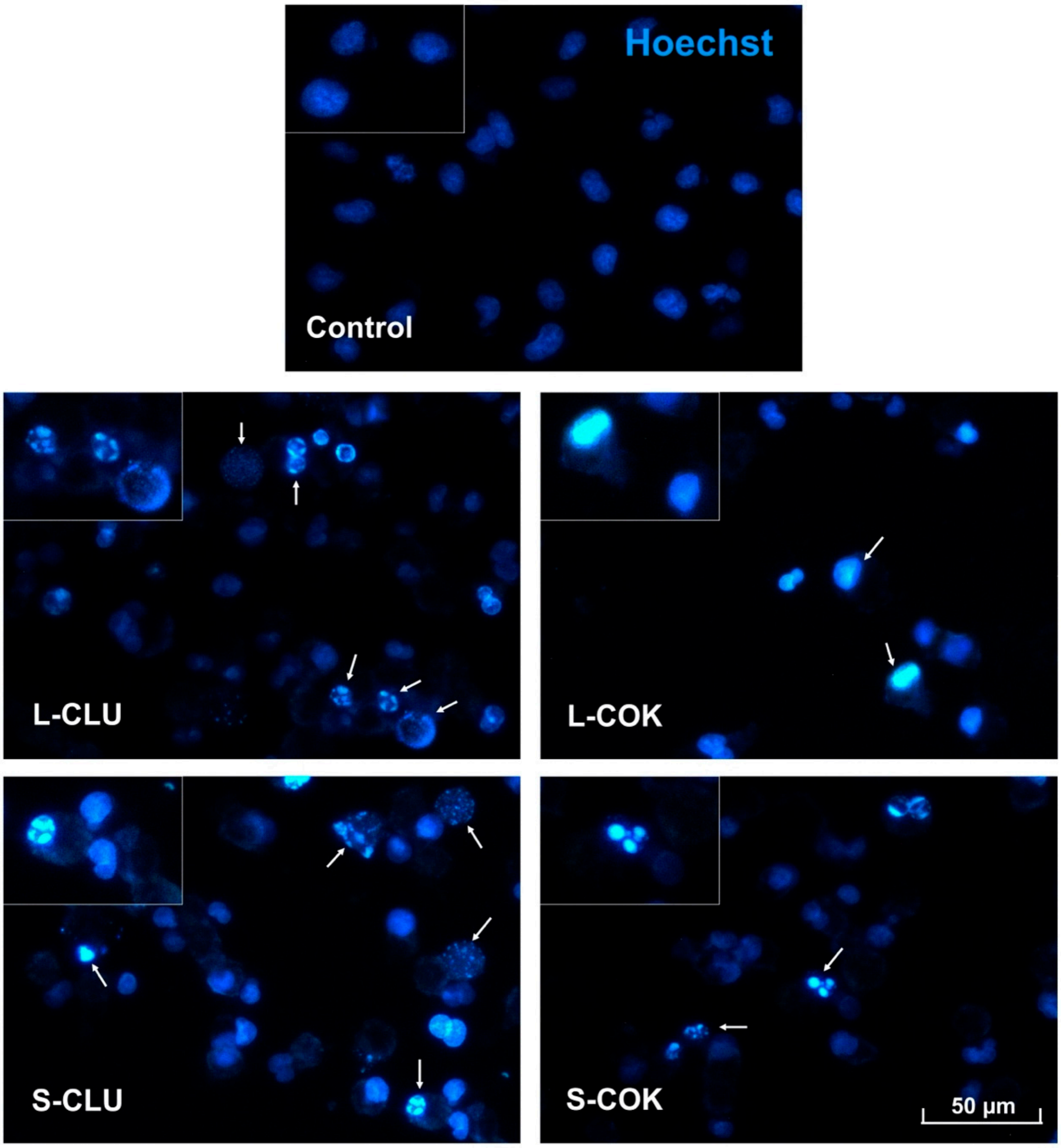


**Figure S5.** Fluorescence images of T84 cells nuclei after treatment with cEtOH extracts from CLU and COK plants. Cells were treated with L- and S-CLU (30 and 40 μg/mL, respectively) and L- and S-COK (30 and 20 μg/mL, respectively) for 24 hours. Nuclei were stained with Hoechst and images were obtained by fluorescence microscopy (amplified from 20× magnification). Arrows indicate apoptotic nuclear morphology including chromatin condensation, nuclear shrinkage and apoptotic bodies.

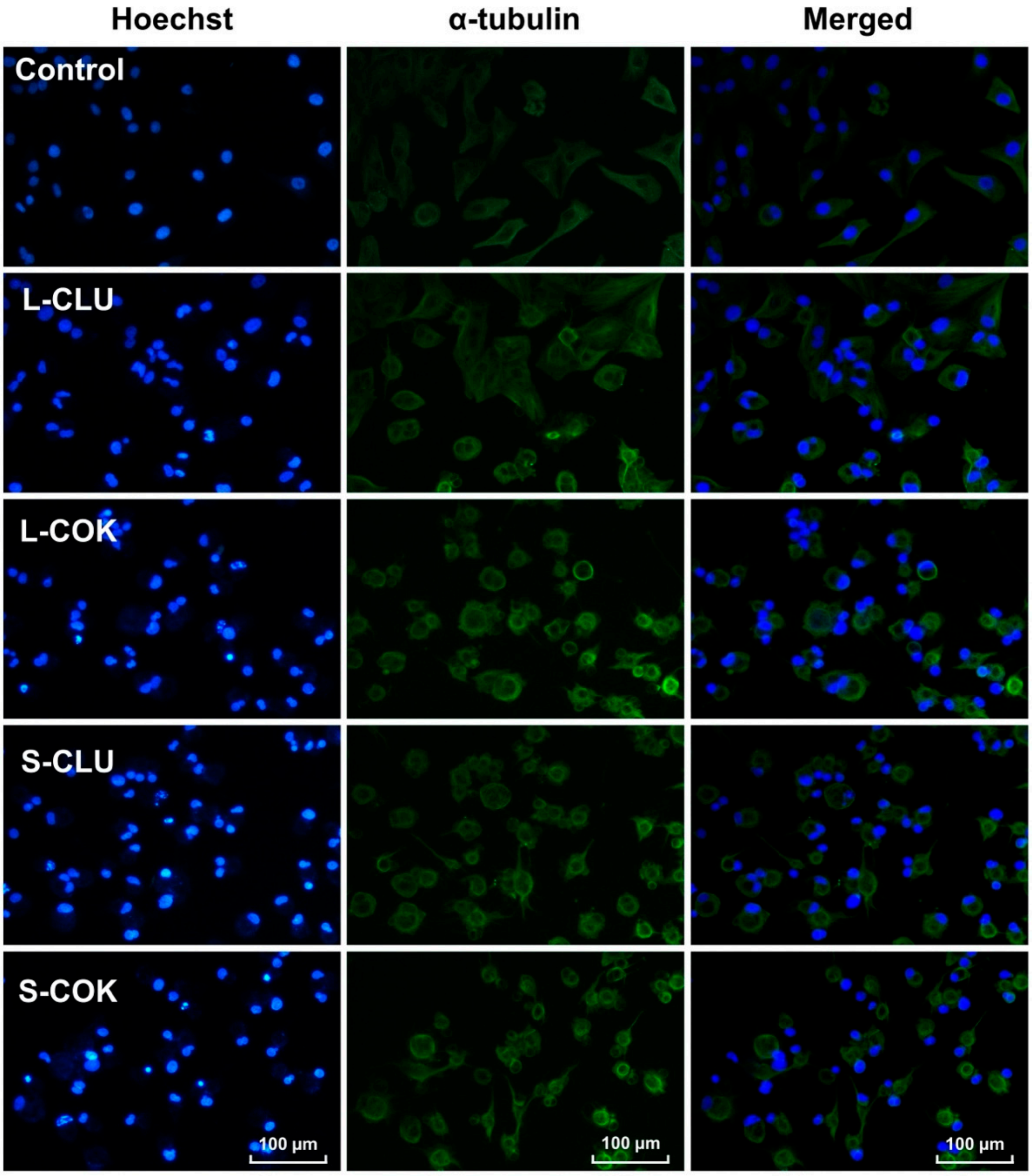


**Figure S6.** Immunofluorescence stain of $\alpha$-tubulin in T84 cells treated with cEtOH from CLU and COK plants. T84 CRC cells were treated with cEtOH from L- and S-CLU (12 and 33 µg/mL, respectively) and L- and S-COK (27 and 12 µg/mL, respectively). $\alpha$-tubulin was stained green and Hoechst 33342 in blue for nuclear localization in fluorescence microscopy images (20× magnification).

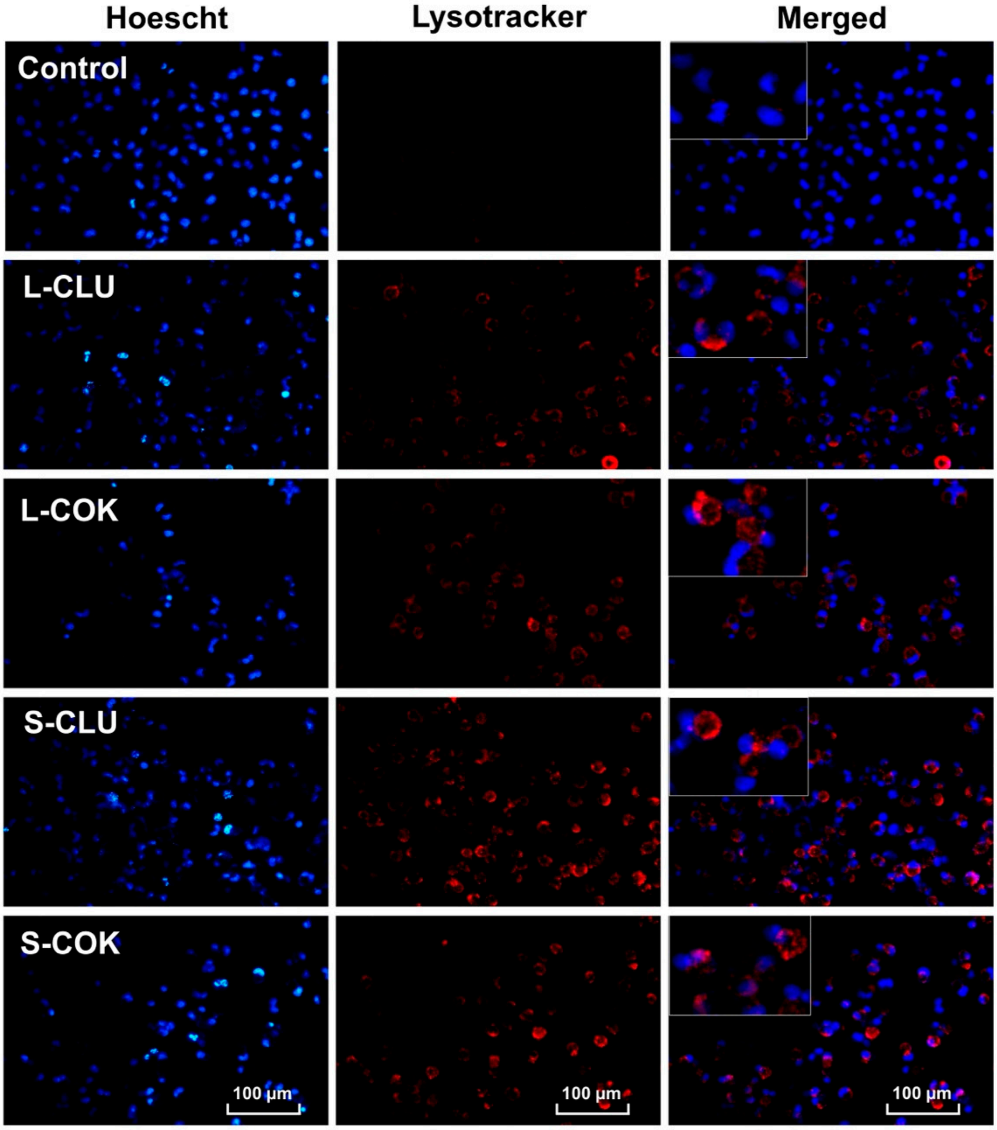


**Figure S7.** Fluorescence detection of autophagic vesicles in T84 cells treated with cEtOH from CLU and COK plants. T84 cells were treated with cEtOH from L- and S-CLU (30 and 40 µg/mL, respectively) and L- and S-COK (30 and 20 µg/mL, respectively) for 24h. The autophagic vesicles were labeled with Lysotracker in red color and the nuclei were labeled with Hoechst 33342 in blue color (20× magnification).

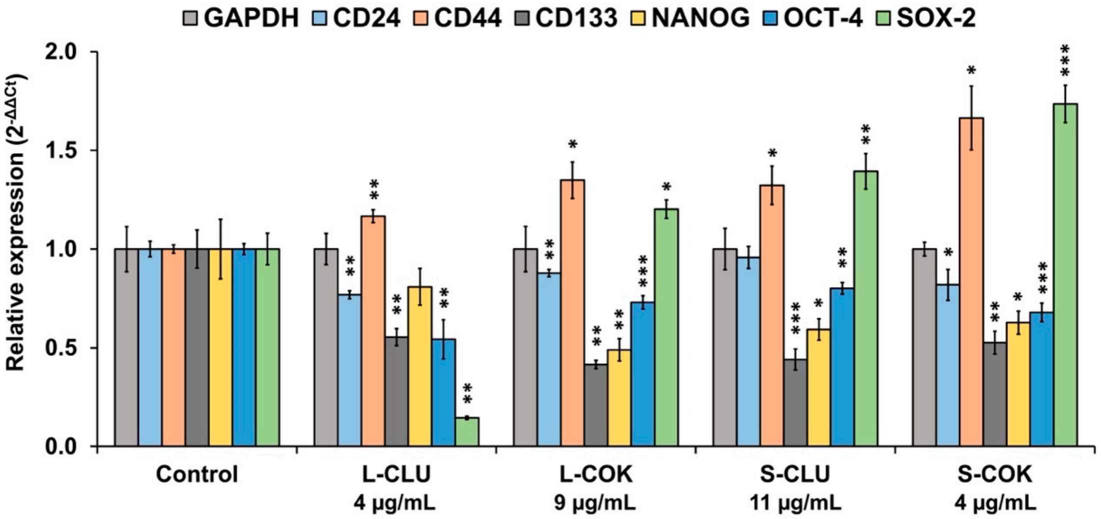


**Figure S8.** CSC-related genes relative expression in T-84 treated with cEtOH extracts from CLU and COK plants. T-84 cells were exposed to $IC_{50}$ of cEtOH from leaves (L) and seeds (S) of CLU and COK for 72 h. Statistical significance compared to control cells: $p$ value < 0.05 (*); < 0.01 (**); < 0.001 (***).

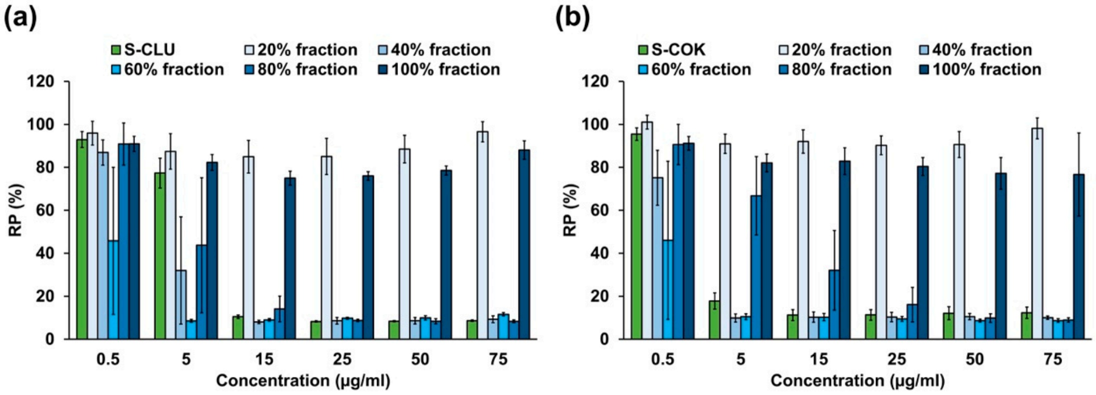


**Figure S9.** Percentage of relative proliferation of T-84 cells treated with fractions from CLU and COK seeds extracts. T84 CRC cells were treated with increasing concentrations of fractions obtained from **(a)** S-CLU and **(b)** S-COK extracts for 72 h. Data were presented as the mean ± SD of 3 replicates from two independent fractionations.

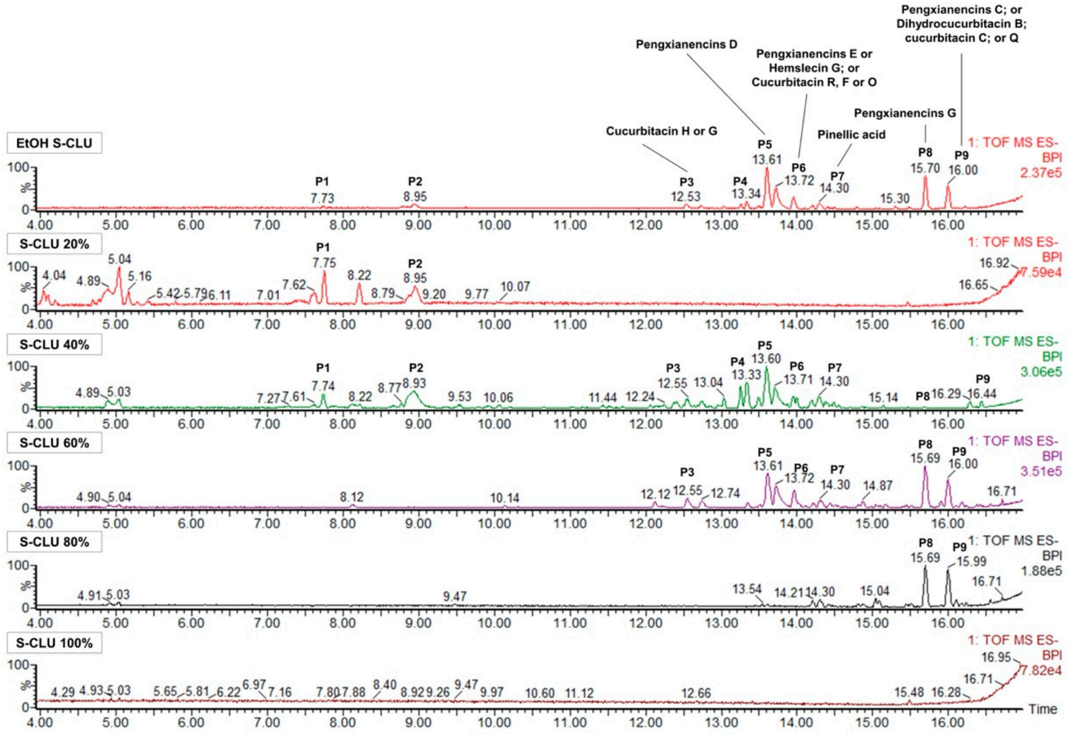


**Figure S10.** UPLC-MS chromatographic profiles of chromatographic fractions obtained from cEtOH of S-CLU. The highest peaks were highlighted and tentatively identified in mass spectrometry databases such as Dictionary of Natural Products and the Chemspider database.

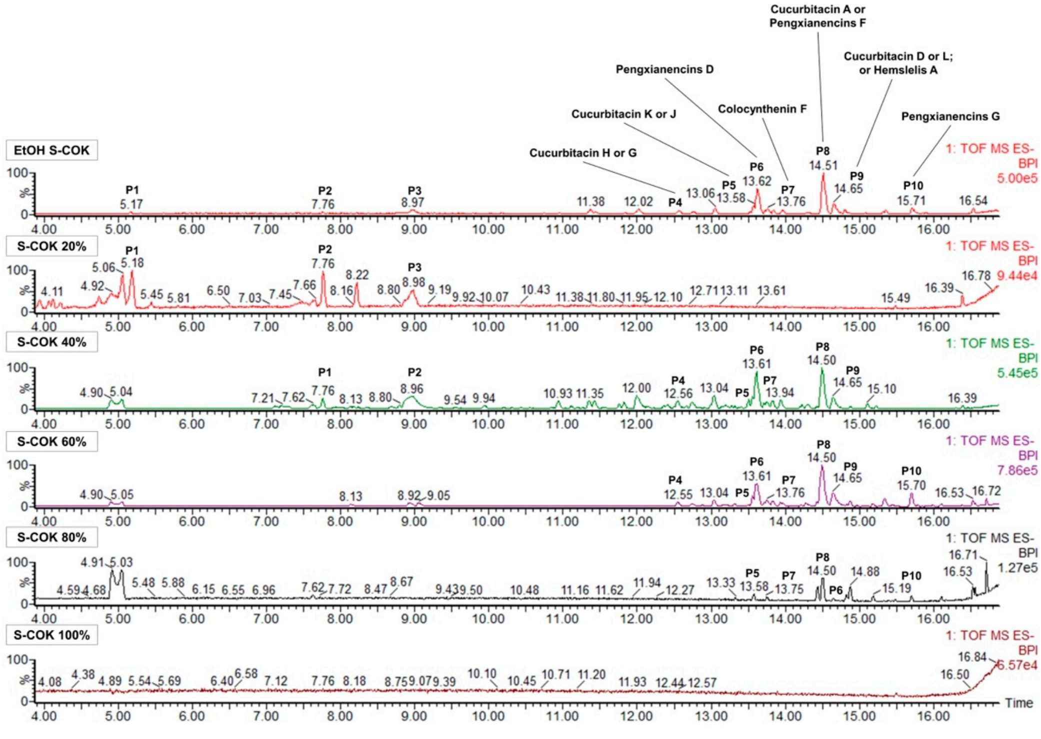


**Figure S11.** UPLC-MS chromatographic profiles of chromatographic fractions obtained from cEtOH of S-COK. The highest peaks were highlighted and tentatively identified in mass spectrometry databases such as Dictionary of Natural Products and the Chemspider database.

**Table S1.** Primary and secondary antibodies used in the western blot experiments

| Type | Target | Origin | Dilution | Commercial reference |
|---|---|---|---|---|
| **Primary antibodies** | Caspase 8 | Mouse | 1:1000 | MA1-41280, Invitrogen |
| | Caspase 9 | Mouse | 1:500 | 9508, Cell Signaling Technology |
| | PARP1 | Rabbit | 1:1000 | ab32138, abcam |
| | VEGFA | Rabbit | 1:1000 | ab46154, abcam |
| | LC3B | Rabbit | 1:1000 | NB100-2220, Novus Biologicals |
| | γ-H2AX (Ser139) | Mouse | 1:1000 | MA12022, Invitrogen |
| **HRP-conjugated IgG secondary antibodies** | Mouse primary antibodies | Horse | 1:2000 | 7076, Cell Signaling Technology |
| | Rabbit primary antibodies | Goat | 1:2000 | 7074, Cell Signaling Technology |
| **Peroxidase antibody** | β-actin | Mouse | 1:25000 | A3854, Sigma Aldrich |

* γ-H2AX (phosphorylated H2A histone family member); HRP (horseradish peroxidase); LC3B (microtubule-associated proteins 1A/1B light chain 3B); PARP1 (poly [ADP-ribose] Polymerase 1); VEGFA (vascular endothelial growth factor A).

**Table S2.** Primers used in the qPCR experiments.

| Gene | Primer | DNA sequence | Tm (°C) |
|---|---|---|---|
| **GAPDH** | Forward | TCGGAGTCAACGGATTTG | 62.4 |
| | Reverse | CAACAATATCCACTTTACCAGAG | 59.1 |
| **CD24** | Forward | CAGTAGTCTTGATGACCAAAG | 56.4 |
| | Reverse | ACAGCATTCTGGAATAAAGC | 58.4 |
| **CD44** | Forward | TTATCAGGAGACCAAGACAC | 56.6 |
| | Reverse | ATCAGCCATTCTGGAATTTG | 61.5 |
| **CD133** | Forward | AAGCATTGGCATCTTCTATG | 59.3 |
| | Reverse | TTTGCTCTGGAGTTTCATTC | 59.4 |
| **OCT-4** | Forward | GATCACCCTGGATATACAC | 58.1 |
| | Reverse | GCTTTGCATATCTCCTGAAG | 59.1 |
| **SOX2** | Forward | ATAATAACAATCATCGGCGG | 61.1 |
| | Reverse | AAAAAGAGAGAGGCAAACTG | 57.8 |
| **NANOG** | Forward | CCAGAACCAGAGAATGAAATC | 60.1 |
| | Reverse | TGGTGGTAGGAAGAGTAAAG | 55.9 |

**Table S3.** Experimental parameters of the two different UPLC-MS analysis.

| Samples | cEtOH L-CLU, L-COK, S-CLU and S-COK | cEtOH S-CLU and S-COK, and their respective fractions |
|---|---|---|
| **UPLC equipment** | ACQUITY UPLC I CLASS (Waters) | ACQUITY UPLC H CLASS (Waters) |
| **Mass Spectrometer** | LCT Premier | QTOF MS SYNAP-G2 (Waters) |
| **Column** | ACQUITY UPLC HSS T3 (100Å, 1.8 μm, 2.1 mm X 100 mm) (Waters) | ACQUITY UPLC BEH Shield RP18 (130Å, 1.7 μm, 2.1 mm X 100 mm) (Waters) |
| **Injection volumn (μL)** | 10 | 10 |
| **Flow rate (mL/min)** | 0.4 | 0.7 |
| **Solvent A** | d$H_2O$ with 0.5% acetic acid | d$H_2O$ with 1% acetic acid |
| **Solvent B** | Acetonitrile | Acetonitrile |
| **Gradient** | Initial - 95% A; 5% B<br>15.00 min - 5% A; 95% B<br>15.10 min - 95% A; 5% B | Initial - 99% A; 1% B<br>2.33 min - 99% A; 1% B<br>4.37 min - 93% A; 7% B<br>8.11 min - 86% A; 14% B<br>12.19 min - 76% A; 24% B<br>15.99 min - 60% A; 40% B<br>18.31 min - 2% A; 98% B<br>21.83 min - 2% A; 98% B<br>22.39 min - 99% A; 1% B<br>25.00 min - 99% A; 1% B |
| **Total run time (min)** | 18 | 28 |
| **Resolution (FWHM)** | 13,500 | 20,000 |
| **ESI Mode** | Negative | Negative |
| **Mass range (Da)** | 100 to 1,200 | 50 to 1,500 |

**Table S4.** UPLC-MS peak tentative identification in cEtOH obtained from leaves and seeds of CLU and COK.

| Peak | Sample | RT (min) | [M-H]- | Error (PPM) | % Conf | Suggested MF | Suggested Compounds | | |
|---|---|---|---|---|---|---|---|---|---|
| | | | | | | | Common Name | MS fragments | Classification |
| P1 | L-CLU | 2.952 | 355.0669 | 1.1 | 99.88 | C15H16O10 | Caffeic acid 3-O-glucuronide | 173.0076; 151.0622; 120.0387 | Phenolic acid / Phenolic Compound |
| | L-CLU | 2.952 | 711.1472 | 4.9 | 97.53 | C30H32O20 | Quercetin 3-O-(6''-malonyl-glucoside) 7-O-glucoside | 306.0469; 273.0515; 173.0071 | Flavonoid / Phenolic Compound |
| P2 | L-CLU | 3.583 | 353.0536 | 7.6 | 99.85 | C15H14O10 | 2-caffeoylisocitric acid | 189.0028; 159.0601; 141.0243 | Phenolic acid; Phenolic Compound |
| | L-COK | 3.582 | 353.0528 | 5.4 | 99.96 | | | 173.0071; 158.9921; 119.0354 | |
| P3 | L-CLU | 4.175 | 337.0545 | -4.5 | 99.96 | C15H14O9 | 7-Hydroxycoumarin glucuronide | 173.0074; 119.0357; 112.9865 | Hydroxycoumarin / Phenolic Compound |
| | L-COK | 4.174 | 337.0539 | -6.2 | 93.05 | | | 306.0468; 273.0532; 173.0078 | |
| P4 | L-CLU | 4.525 | 367.0635 | -8.2 | 99.33 | C16H16O10 | Scopoletin b-D-glucuronide | 173.0085; 119.0357; 112.9864 | Hydroxycoumarin / Phenolic Compound |
| | L-COK | 4.49 | 367.0626 | -10.6 | 97.55 | | | 306.0464; 219.0862; 173.0067 | |
| | L-CLU | 4.525 | 463.0882 | 1.1 | 99.5 | C21H20O12 | Isoquercetin | 173.0085; 119.0357; 112.9864 | Flavonoid /Phenolic Compound |
| P5 | L-CLU | 4.773 | 549.0915 | 6.4 | 99.12 | C24H22O15 | Quercetin 3-O-malonylglucoside | 273.0546; 173.0088; 119.0357 | Flavonoid / Phenolic Compound |
| | L-COK | 4.771 | 549.0894 | 2.5 | 98.8 | | | 306.0468; 219.0864; 173.0065 | |
| P6 | L-CLU | 5.015 | 447.0925 | -0.4 | 99.69 | C21H20O11 | Quercitrin | 306.0462; 173.0081; 119.0358 | Flavonoid / Phenolic Compound |
| | L-COK | 5.014 | 447.0933 | 1.3 | 98.27 | | | 306.0451; 219.0865; 173.0071 | |

| Peak | Sample | RT (min) | [M-H]- | Error (PPM) | % Conf | Suggested MF | Suggested Compounds | | |
|---|---|---|---|---|---|---|---|---|---|
| | | | | | | | Common Name | MS fragments | Classification |
| P7 | L-CLU | 5.399 | 563.1028 | -1.6 | 99.61 | C25H24O15 | Isorhamnetin 3-(6''-malonylglucoside) | 306.0459; 219.0880; 173.0081 | Flavonoid / Phenolic Compound |
| | L-COK | 5.363 | 563.1022 | -2.7 | 99.24 | | | 306.0462; 173.0836; 119.0350 | |
| P8 | L-CLU | 7.253 | 327.2161 | -3.1 | 99.66 | C18H32O5 | 12,13,16-Trihydroxy-9,14-octadecadienoic acid derivates/ 12,15,16-Trihydroxy-9,13-octadecadienoic acid derivates | - | Oxylipins |
| | L-COK | 7.218 | 327.2176 | 1.5 | 99.81 | | | - | |
| | S-CLU | 7.16 | 327.2169 | -0.6 | 99.49 | | | - | |
| P9 | L-CLU | 7.569 | 779.3893 | 5 | 92.61 | C40H60O15 | - | - | Steroids |
| P10 | L-CLU | 8.86 | 589.3236 | 2 | 99.05 | C29H50O12 | - | - | Carbohydrate |
| P11 | L-CLU | 9.525 | 617.3359 | 5.3 | 99.12 | C34H50O10 | Pengxianencins G | N.A. | Cucurbitane-type triterpenoid |
| | L-COK | 9.525 | 617.3344 | 2.9 | 99.25 | | | N.A. | |
| | S-CLU | 9.262 | 617.3356 | 4.9 | 76.73 | | | N.A. | |
| | S-COK | 9.257 | 617.3352 | 4.2 | 98.57 | | | N.A. | |
| P12 | L-CLU | 10.19 | 615.3182 | 2.1 | 99.55 | C34H48O10 | - | - | Terpenoid |
| | L-COK | 10.19 | 615.3182 | 2.1 | 99.4 | | | - | |
| | S-COK | 9.92 | 615.319 | 3.4 | 99.12 | | | - | |
| P13 | L-CLU | 11.098 | 899.5381 | 1.4 | 86.39 | C47H80O16 | Dammar-24-ene-3,20,21-triol; (3β,20S)-form; 3-O-[α-L-Rhamnopyranosyl-(1→2)-[β-D-xylopyranosyl-(1→3)]-β-D-glucopyranoside] | N.A. | Dammarane-type triterpenoid |
| | L-COK | 11.064 | 899.5405 | 4.1 | 85.86 | | | N.A. | |
| P14 | L-CLU | 11.341 | 587.4313 | 0.2 | 99.49 | C36H60O6 | 26,27-Dimethyl-26-methylenecholest-5-en-3-ol; (3β,25ξ)-form, 3-O-β-D-Glucopyranoside | N.A. | Cholestane steroid |
| | L-COK | 11.34 | 587.429 | -3.7 | 90.4 | | | N.A. | |
| | S-CLU | 11.26 | 587.4299 | -2.2 | 91.25 | | | N.A. | |
| | S-COK | 11.11 | 587.4312 | 0 | 97.02 | | | N.A. | |
| P15 | L-CLU | 11.656 | 1087.6431 | -1.2 | 99.27 | C29H50O11 | - | - | - |

| Peak | Sample | RT (min) | [M-H]- | Error (PPM) | % Conf | Suggested MF | Suggested Compounds | | |
|---|---|---|---|---|---|---|---|---|---|
| | | | | | | | Common Name | MS fragments | Classification |
| | L-COK | 11.656 | 573.3294 | 3.3 | 99.83 | | | - | |
| P16 | L-CLU | 12.461 | 431.2215 | -15.3 | 83.11 | C21H36O9 | - | - | Terpenoid |
| | L-COK | 12.738 | 431.2196 | -19.7 | 70.35 | | | - | |
| P17 | L-CLU | 13.51 | 411.2747 | 0.0 | 99.7 | C23H40O6 | - | - | Terpenoid |
| | L-COK | 13.51 | 411.2748 | 0.2 | 99.92 | | | - | |
| P18 | L-CLU | 14.209 | 865.5347 | 3.9 | 88.46 | C47H78O14 | - | - | - |
| P19 | L-CLU | 14.384 | 417.3212 | -1 | 99.9 | C23H46O6 | - | - | Aliphatic natural product |
| | L-COK | 14.417 | 417.3209 | -1.7 | 99.73 | | | - | |
| | S-COK | 13.81 | 417.3196 | -4.8 | 97.93 | | | - | |
| P20 | L-CLU | 14.593 | 555.442 | 1.3 | 99.65 | C36H60O4 | - | - | - |
| | L-COK | 14.592 | 555.442 | 1.3 | 94.63 | | | - | |
| P21 | L-COK | 6.17 | 765.2615 | 1.2 | 98 | C36H46O18 | - | - | - |
| P22 | L-COK | 7.393 | 655.4454 | 5 | 98.06 | C36H64O10 | - | - | Dammarane-type triterpenoid |
| P23 | L-COK | 8.369 | 573.3074 | 1.7 | 99.68 | C32H46O9 | Cucurbitacin A / Pengxianencins F | (559.2914; 293.1777; 159.0620) / N.A. | Cucurbitane-type triterpenoid |
| | S-COK | 8.11 | 573.3056 | -1.4 | 99.97 | | | (560.2944; 559.2905; 515.2968) / N.A. | |
| P24 | L-COK | 10.083 | 619.4231 | 3.4 | 98.98 | C36H60O8 | Balsaminoside B | (389.2831; 353.2831; 119.0356) | Cucurbitane-type triterpenoid |
| P25 | S-CLU | 4.6 | 733.3333 | 6.8 | 85.85 | C34H54O17 | - | - | - |
| | S-COK | 4.6 | 733.3306 | 3.1 | 70.26 | | | - | |
| P26 | S-CLU | 5.86 | 979.4788 | 3.9 | 73.81 | C46H76O22 | - | - | - |
| P27 | S-CLU | 6.04 | 981.4936 | 3.1 | 89.66 | C46H78O22 | - | - | - |
| P28 | S-CLU | 6.71 | 593.3326 | 0 | 96.19 | C32H50O10 | - | - | - |
| | S-COK | 6.81 | 593.332 | -1 | 99.17 | | | - | |
| P29 | S-CLU | 6.92 | 493.2078 | 0.8 | 99.76 | C25H34O10 | - | - | - |

| Peak | Sample | RT (min) | [M-H]- | Error (PPM) | % Conf | Suggested MF | Suggested Compounds | | |
|---|---|---|---|---|---|---|---|---|---|
| | | | | | | | Common Name | MS fragments | Classification |
| P30 | S-CLU | 7.509 | 575.3215 | -0.9 | 97.65 | C32H48O9 | Pengxianencins D/ 23,24-dihydrocucurbitacin A / 7-hydroxycucurbitacin F-25-O-acetate | N.A. / (400.2678) / (400.2678) | Cucurbitane-type triterpenoid |
| | S-COK | 7.507 | 575.3212 | -1.4 | 99.74 | | | N.A. / (414.2766; 400.2586; 196.0661) / (414.2766; 400.2586; 196.0661) | |
| P31 | S-CLU | 8.28 | 493.3166 | 0.2 | 99.71 | C28H46O7 | - | - | - |
| P32 | S-CLU | 9.51 | 619.3475 | 8.4 | 96.97 | C41H48O5 | - | - | - |
| P33 | S-CLU | 11.96 | 295.2278 | 1.7 | N.A. | C18H32O3 | 18-Hydroxylinoleic acid | - | Fatty acid |
| | S-COK | 11.96 | 295.2254 | -6.4 | N.A. | | | - | |
| P34 | S-CLU | 12.41 | 297.243 | 0 | N.A. | C18H34O3 | Ricinoleic acid | - | Fatty acid |
| | S-COK | 12.41 | 297.2425 | -1.7 | N.A. | | | - | |
| P35 | S-CLU | 14.24 | 413.2883 | -4.8 | 87.75 | C23H42O6 | - | - | - |
| | S-COK | 14.23 | 413.2906 | 0.7 | 99.96 | | | - | |
| P36 | S-CLU | 15.21 | 609.5109 | 2.5 | 98.43 | C37H70O6 | - | - | - |
| | S-COK | 15.21 | 609.51 | 1 | 99.1 | | | - | |
| P37 | S-COK | 5.83 | 737.3768 | 2.7 | 53.78 | C38H58O14 | - | - | Steroid |
| P38 | S-COK | 6.11 | 607.3118 | 0 | 98.16 | C32H48O11 | - | - | Steroid |
| P39 | S-COK | 7.06 | 545.2748 | -0.6 | 99.3 | C30H42O9 | 16,20,25-Trihydroxy-11,22-dioxo-2,3-secocucurbita-1(10),5,23-triene-2,3-dioic acid | N.A. | Cucurbitane-type triterpenoid |
| P40 | S-COK | 7.41 | 591.3184 | 2.5 | 97.5 | C32H48O10 | Colocynthenin F | 220.0325 | Cucurbitane-type triterpenoid |
| P41 | S-COK | 9.155 | 619.3472 | -1.6 | 96.81 | C34H52O10 | Pengxianencins C | N.A. | Cucurbitane-type triterpenoid |

(*) Not available data (N.A.)

**Table S5**. UPLC-MS peak tentative identification in fractions obtained from cEtOH of S-CLU.

| Peak | Sample | RT (min) | [M-H]- | Error (PPM) | % Conf | Suggested MF | Suggested Compounds | | |
|---|---|---|---|---|---|---|---|---|---|
| | | | | | | | Common Name | MS Fragments | Classification |
| 1 | EtOH S-CLU | 7.73 | 776.3096 | - | - | - | - | - | - |
| | S-CLU 20% | 7.759 | 776.3135 | - | - | | | - | |
| | S-CLU 40% | 7.737 | 776.3137 | - | - | | | - | |
| 2 | EtOH S-CLU | 8.965 | 673.3038 | -4.9 | 99.69 | C32H50O15 | - | - | - |
| | S-CLU 20% | 8.950 | 673.3049 | -3.3 | 85.53 | | | - | |
| | S-CLU 40% | 8.927 | 673.3045 | -3.9 | 99.99 | | | - | |
| 3 | EtOH S-CLU | 12.544 | 533.3116 | 0.4 | 84.81 | C30H46O8 | Cucurbitacin H / G | (428.2518; 427.2499; 341.2148) | Cucurbitane-type triterpenoid |
| | S-CLU 40% | 12.551 | 533.313 | 3 | 99.98 | | | (445.2607; 427.2494; 341.2145) | |
| | S-CLU 60% | 12.551 | 533.3122 | 1.5 | 99.75 | | | (515.3002; 445.2598; 427.2492) | |
| 4 | EtOH S-CLU | 13.345 | 493.2069 | -1 | 98.45 | C25H34O10 | - | - | Terpenoid |
| | S-CLU 40% | 13.337 | 493.2083 | 1.8 | 100 | | | - | |
| 5 | EtOH S-CLU | 13.607 | 575.3214 | -1 | 99.79 | C32H48O9 | Pengxianencins D / 23,24-dihydrocucurbitacin A / 7-hydroxycucurbitacin F-25-O-acetate | N.A. / (515.2995; 497.2920; 327.2170) / (515.2995; 497.2920; 327.2170) | Cucurbitane-type triterpenoid |
| | S-CLU 40% | 13.599 | 575.3225 | 0.9 | 100 | | | N.A. / (515.3018; 497.2920; 328.2229) / (515.3018; 497.2920; 328.2229) | |
| | S-CLU 60% | 13.614 | 575.3198 | -3.8 | 97.92 | | | N.A. / (515.2999; 497.2805; 327.2184) / (515.2999; 497.2805; 327.2184) | |
| 6 | EtOH S-CLU | 13.719 | 577.3374 | -0.5 | 99.92 | C32H50O9 | Pengxianencins E / Hemslecin G | N.A. | Cucurbitane-type triterpenoid |
| | S-CLU 40% | 13.711 | 577.3371 | -1 | 99.96 | | | N.A. | |
| | S-CLU 60% | 13.726 | 577.3345 | -5.5 | 99.99 | | | N.A. | |
| | EtOH S-CLU | 13.719 | 517.3165 | 0 | 99.34 | C30H46O7 | | (499.3076; 385.2386; 327.2186) | |

| | | | | | | | | | |
|---|---|---|---|---|---|---|---|---|---|
| | S-CLU 40% | 13.711 | 517.317 | 1 | 90.74 | | Cucurbitacin R / Cucurbitacin F / Cucurbitacin O | (499.3063; 385.2375; 328.2213) | Cucurbitane-type triterpenoid |
| | S-CLU 60% | 13.726 | 517.3149 | -3.1 | 99.99 | | | (499.3033; 385.2363; 327.2168) | |
| 7 | EtOH S-CLU | 14.295 | 329.2348 | 6.1 | N.A. | C18H34O5 | Pinellic acid | (211.1336; 171.1048; 127.1117) | Fatty acid |
| | S-CLU 40% | 14.295 | 329.2342 | 4.3 | N.A. | | | (229.1462; 211.1358; 171.1039) | |
| | S-CLU 60% | 14.31 | 329.2346 | 5.5 | N.A. | | | (229.1459; 211.1359; 171.1048) | |
| | S-CLU 80% | 14.302 | 329.233 | 0.6 | 99.59 | | | (293.2141; 211.1337; 171.1041) | |
| 8 | EtOH S-CLU | 15.695 | 617.3322 | -0.6 | 99.99 | C34H50O10 | Pengxianencins G | N.A. | Cucurbitane-type triterpenoid |
| | S-CLU 40% | 15.68 | 617.3322 | -0.6 | 99.86 | | | N.A. | |
| | S-CLU 60% | 15.695 | 617.332 | -1 | 99.95 | | | N.A. | |
| | S-CLU 80% | 15.694 | 617.33 | -4.2 | 99.95 | | | N.A. | |
| 9 | EtOH S-CLU | 16.002 | 619.3452 | -4.8 | 94.01 | C34H52O10 | Pengxianencins C | N.A. | Cucurbitane-type triterpenoid |
| | S-CLU 60% | 16.002 | 619.3483 | 0.2 | 99.99 | | | N.A. | |
| | S-CLU 80% | 16.001 | 619.3455 | -4.4 | 99.79 | | | N.A. | |
| | EtOH S-CLU | 16.002 | 559.3259 | -2.1 | 99.95 | C32H48O8 | Dihydrocucurbitacin B / Cucurbitacin C / Cucurbitacin Q | (481.2954; 412.2264; 341.2119) | Cucurbitane-type triterpenoid |
| | S-CLU 40% | 16.443 | 559.3265 | -1.1 | 99.56 | | | (559.3331; 481.2941) | |
| | S-CLU 60% | 16.002 | 559.3284 | 2.3 | 99.92 | | | (481.2971; 412.2282; 341.2150) | |
| | S-CLU 80% | 16.001 | 559.3251 | -3.6 | 96.33 | | | (481.2954; 412.2262; 341.2122) | |

(*) Not available data (N.A.)

**Table S6**. UPLC-MS peak tentative identification in fractions obtained from cEtOH of S-COK.

| Peak | Sample | RT (min) | [M-H]- | Error (PPM) | % Conf | Suggested MF | Suggested Compounds | | |
|---|---|---|---|---|---|---|---|---|---|
| | | | | | | | Common Name | MS fragments | Classification |
| 1 | EtOH S-COK | 5.162 | 475.1822 | 1.3 | 99.41 | C21H32O12 | - | - | - |
| | S-COK 20% | 5.185 | 475.181 | -1.3 | 100 | | | - | - |
| 2 | EtOH S-COK | 7.759 | 776.314 | - | - | - | - | - | - |
| | S-COK 20% | 7.775 | 776.3128 | - | - | | | - | - |
| | S-COK 40% | 7.759 | 776.3124 | - | - | | | - | - |
| 3 | EtOH S-COK | 8.972 | 673.306 | -1.6 | 100 | C32H50O15 | - | - | - |
| | S-COK 20% | 8.973 | 673.3066 | -0.7 | 89.96 | | | - | - |
| | S-COK 40% | 8.973 | 673.3087 | 2.4 | 99.08 | | | - | - |
| 4 | EtOH S-COK | 12.56 | 533.3122 | 1.5 | 99.97 | C30H46O8 | Cucurbitacin H / G | (445.2613; 427.2491; 341.2132) | Cucurbitane-type triterpenoid |
| | S-COK 40% | 12.55 | 533.3112 | -0.2 | 99.78 | | | (445.2601; 427.2484; 341.2130) | |
| | S-COK 60% | 12.54 | 533.3101 | -2.4 | 99.98 | | | (445.2578; 427.2479; 341.2120) | |
| 5 | EtOH S-COK | 13.57 | 531.2959 | 0.2 | 99.24 | C30H44O8 | Cucurbitacin K / Cucurbitacin J | (443.2434; 425.2333; 383.2216) | Cucurbitane-type triterpenoid |
| | S-COK 40% | 13.55 | 531.295 | -1.5 | 96.7 | | | (443.2439; 425.2325; 328.2217) | |
| | S-COK 60% | 13.56 | 531.2961 | 0.6 | 100 | | | (443.2434; 425.2325; 327.2181) | |
| | S-COK 80% | 13.58 | 531.295 | -1.5 | 97.87 | | | (426.2373; 425.2333; 339.1988) | |
| 6 | EtOH S-COK | 13.63 | 575.3215 | -0.9 | 99.09 | C32H48O9 | Pengxianencins D / 23,24-dihydrocucurbitacin A / 7-hydroxycucurbitacin F-25-O-acetate | N.A. / (515.3011; 425.2340; 327.2191) / (515.3011; 479.2796; 327.2191) | Cucurbitane-type triterpenoid |
| | S-COK 40% | 13.61 | 575.3214 | -1 | 99.52 | | | N.A. / (515.3015; 479.2809; 327.2184) / (515.3015; 479.2809; 327.2184) | |

| | | | | | | | | | |
|---|---|---|---|---|---|---|---|---|---|
| | S-COK 60% | 13.62 | 575.3215 | -0.9 | 99.97 | | | N.A. / (515.3008; 425.2322; 327.2174) / (515.3008; 479.2795; 327.2174) | |
| | S-COK 80% | 14.65 | 575.3215 | -0.9 | 98.18 | | | N.A. / (426.2336; 425.2316; 339.1933) | |
| 7 | EtOH S-COK | 13.76 | 591.3163 | -1 | 91.25 | C32H48O10 | Colocynthenin F | (513.2854; 443.2435; 425.2327) | Cucurbitane-type triterpenoid |
| | S-COK 60% | 13.76 | 591.3173 | 0.7 | 99.63 | | | (531.2969; 443.2447; 425.2335) | |
| | S-COK 80% | 13.75 | 591.3159 | -1.7 | 97.6 | | | (513.2862; 443.2451; 425.2329) | |
| 8 | EtOH S-COK | 14.51 | 573.3066 | 0.3 | 99.97 | C32H46O9 | Cucurbitacin A / Pengxianencins F | (513.2863; 495.2759; 477.2650) / N.A. | Cucurbitane-type triterpenoid |
| | S-COK 40% | 14.5 | 573.3052 | -1.2 | 99.89 | | | (513.2848; 495.2741; 477.2631) / N.A. | |
| | S-COK 60% | 14.48 | 573.3055 | -1.6 | 99.93 | | | (513.2850; 477.2639; 383.2224) / N.A. | |
| | S-COK 80% | 14.5 | 573.3055 | -1.6 | 20.72 | | | (513.2883; 495.2753; 435.2565) / N.A. | |
| 9 | EtOH S-COK | 14.65 | 515.3009 | 0 | 99.3 | C30H44O7 | Cucurbitacin D / Cucurbitacin L | (497.2910; 384.2258; 383.2221) / (455.2805; 383.2221; 339.1972) | Cucurbitane-type triterpenoid |
| | S-COK 40% | 14.65 | 515.3011 | 0.4 | 99.99 | | | (497.2896; 383.2224; 325.2030) / (455.2792; 383.2224; 325.2030) | |
| | S-COK 60% | 14.65 | 515.2999 | -1.9 | 99.6 | | | (497.2893; 455.2798; 383.2224) / (455.2798; 383.2224; 327.2182) | |
| 10 | EtOH S-COK | 15.72 | 617.3306 | -3.2 | 48.44 | C34H50O10 | Pengxianencins G | N.A. | Cucurbitane-type triterpenoid |
| | S-COK 60% | 15.7 | 617.3306 | -3.2 | 99.82 | | | N.A. | |
| | S-COK 80% | 15.7 | 617.3329 | 0.5 | 79.17 | | | N.A. | |

(*) Not available data (N.A.)
